# UV-irradiation of self-assembled triphenylamines affords persistent and regenerable radicals†
†Electronic supplementary information (ESI) available: Experimental details; synthesis and characterization of compounds; SC-XRD data; PXRD, absorbance emission, DOSY NMR, EPR, ^1^H NMR, FTIR, CV, and electrolysis spectra; and luminescent lifetime calculations and spectra. CCDC 1873066. For ESI and crystallographic data in CIF or other electronic format see DOI: 10.1039/c8sc04607g


**DOI:** 10.1039/c8sc04607g

**Published:** 2019-01-10

**Authors:** Ammon J. Sindt, Baillie A. DeHaven, David F. McEachern, D. M. M. Mevan Dissanayake, Mark D. Smith, Aaron K. Vannucci, Linda S. Shimizu

**Affiliations:** a Department of Chemistry and Biochemistry , University of South Carolina , Columbia , South Carolina 29208 , USA . Email: SHIMIZLS@mailbox.sc.edu

## Abstract

UV-irradiation of assembled urea-tethered triphenylamine dimers results in the formation of persistent radicals, whereas radicals generated in solution are reactive and quickly degrade. In the solid-state, high quantities of radicals (approximately 1 in 150 molecules) are formed with a half-life of one week with no significant change in the single crystal X-ray diffraction. Remarkably, after decay, re-irradiation of the solid sample regenerates the radicals to their original concentration. The photophysics upon radical generation are also altered. Both the absorption and emission are significantly quenched without external oxidation likely due to the delocalization of the radicals within the crystals. The factors that influence radical stability and generation are correlated to the rigid supramolecular framework formed by the urea tether of the triphenylamine dimer. Electrochemical evidence demonstrates that these compounds can be oxidized in solution at 1.0 V *vs.* SCE to generate radical cations, whose EPR spectra were compared with spectra of the solid-state photogenerated radicals. Additionally, these compounds display changes in emission due to solvent effects from fluorescence to phosphorescence. Understanding how solid-state assembly alters the photophysical properties of triphenylamines could lead to further applications of these compounds for magnetic and conductive materials.

## Abstract 2

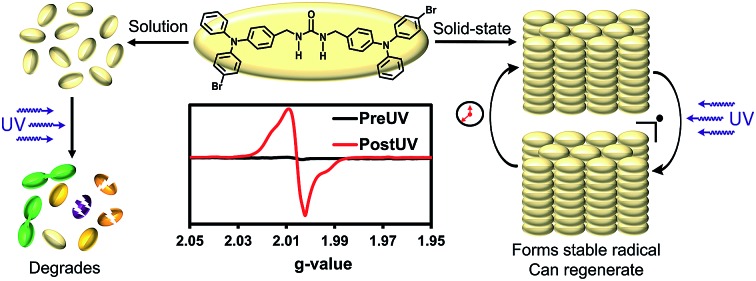
UV-irradiation of assembled urea-tethered triphenylamine dimers affords persistent and regenerable radicals whereas the compound quickly degrades in solution.

## Introduction

**Fig. 1:**
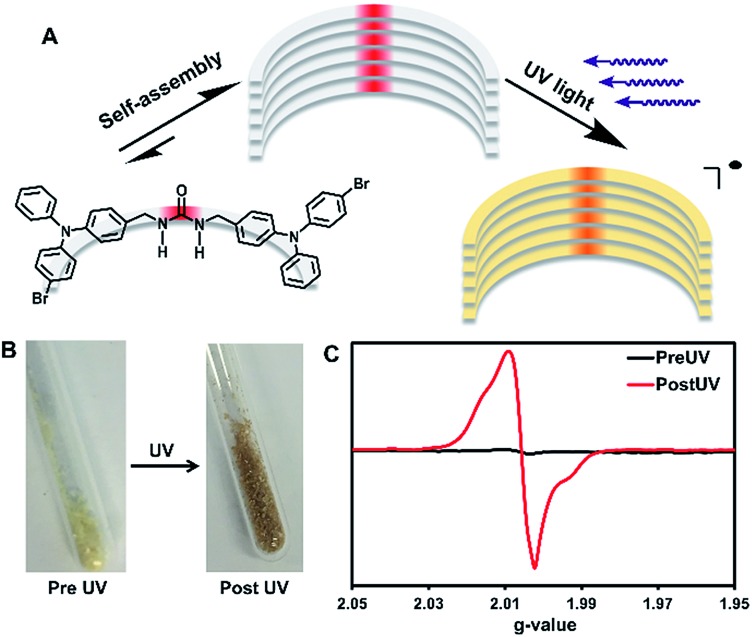
(A) Self-assembly of a triphenylamine derivative affords persistent radicals upon irradiation with UV light. (B) UV-irradiation induces a noticeable change in color. (C) A significant radical signal is observed which corresponds to 1 in ∼600 molecules displaying a radical after 1 h of UV-irradiation and up to 1 in ∼150 after 8–11 h.

Intentional design of functional supramolecular assemblies requires precise control of intermolecular interactions as well as an understanding of how complex structures modulate chemical and physical properties to produce materials with emergent qualities.^1^ This understanding is key for designing compounds used to probe the enhancement or quenching of luminescence of small molecules in the solid-state.^2^ Controlled assembly of structures can also modulate conductivity^3^ and dichroism in photoactive materials.^4^ Here, we synthesize urea tethered triphenylamines (TPAs), and determine their photophysical properties in solution and in crystalline assemblies. Upon UV-irradiation, in both solution and the solid-state, these materials displayed radical formation with solid-state samples proving to be quite stable (Fig. 1). Remarkably, solid-state samples yield high quantities of persistent radicals with ∼1 in 150 molecules containing a radical. Moreover, after decay, re-irradiation with UV light can regenerate the radicals in similar quantities. Thus, solid-state assembly alters the photophysical properties of TPAs and could prove helpful in the design of conductive and magnetic materials that integrate TPA components.

*Para* substituted TPAs are prevalent examples of molecules that exhibit persistent radicals.^5^ For example, Magic Blue, an antimony salt of tribromo TPA, is a commercial one-electron oxidant employed for many chemical processes.^6^ The stability of substituted TPA radicals, has led to their use as promising spin-containing units for organic polymer based magnets.^7^ These organic magnets are designable offering moldability and tunability. The oxidation of the TPA also alters its photophysics, leading to quenching of its fluorescence.^8^ Typically, TPA compounds require *para* substitution on all the phenyl rings to generate stable radical cations.^9^,^10^ The extra substitution helps to slow down degradation reactions such as benzidine formation.^9^ Usually, chemical or electrochemical oxidation is required to generate the radicals.^8^ Even without the oxidation to a radical, TPAs still find many uses as two-photon absorbers,^11^ organic light emitting diode materials,^12^ solvatofluorochromatic intramolecular charge transfer (ICT) molecules,^13^ and as aggregation induced emission (AIE) compounds.^14^

The Shimizu group utilizes the three-centered urea interaction to drive assembly of linear and macrocyclic monomers into tapes, rods, and columns.^15^ In the case of benzophenone containing monomers, assembly influences the photophysics and affords surprisingly stable radicals upon UV-irradiation.^16^ For comparison, unassembled structures in solution show no radical formation upon UV-irradiation. Our hypothesis is that supramolecular assembly significantly enhances radical stability. Here, we test if urea-tethered triphenylamines will be affected in a similar manner.

We synthesized and compared the structures and properties of a methylene urea bridged 4-bromo TPA dimer (**3**) against 4-bromo TPA (**1**) and a protected urea analog (**2**). The structure of dimer **3** features one bromine on each TPA adduct to assist in intersystem crossing (ISC) from the excited singlet to the triplet state which can be aided by the heavy atom effect, and should help promote radical generation from UV-irradiation. The heavy atom effect increases ISC due to spin orbit coupling.^17^ Additionally, one *para* position on each TPA unit of **3** was left intentionally unsubstituted. Typically, fully substituted TPA's are required for radical stability.^5^ Here, we test if supramolecular assembly can provide stability to unsubstituted TPA radicals, which in turn, would allow for greater variability in TPA structures with stable radical characteristics. Radical formation was investigated by two methods: electrochemical oxidation and UV-irradiation. Both of these methods can generate radical cations in TPA compounds, with the former being well-known,^8^ and the latter requiring a reducible agent in the molecule itself^18^ or in molecules close-by (*i.e.* solvent).^19^ Our goal is to characterize these systems by electron paramagnetic resonance (EPR) spectroscopy to understand how solid-state organization influences their ability to generate stable radicals *versus* dissolution. Specifically, we are testing (1) if self-assembly can stabilize radicals and (2) if UV-irradiation is a useful tool to generate TPA radicals in reasonable quantities. Additionally, we will examine the solvent dependent photophysics of the triplet and singlet emissions of these molecules.

## Results and discussion

**Scheme 1:**
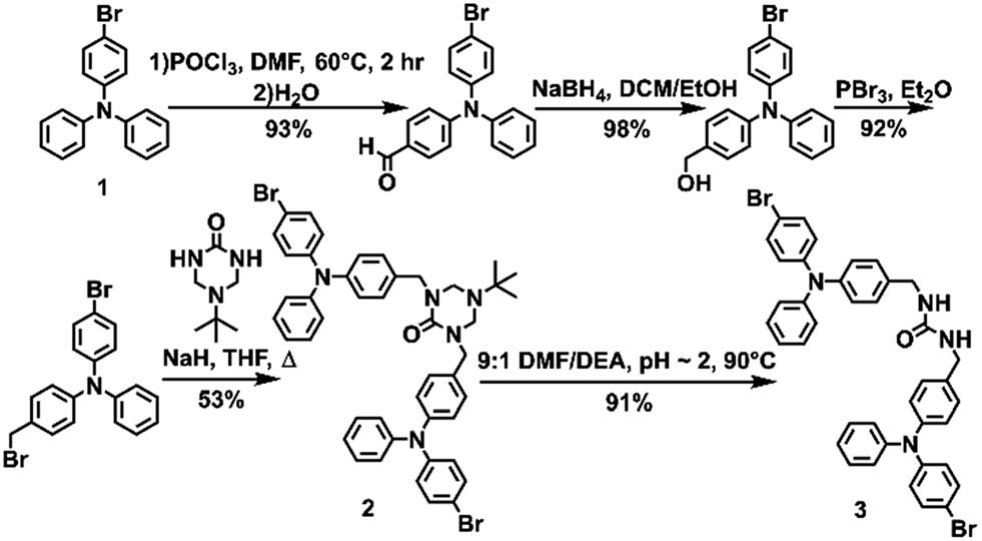
Synthetic scheme for **2** and **3**.

The urea tethered triphenylamine **3** was synthesized in five steps from commercial 4-bromotriphenylamine using a Vilsmeier–Haack reaction with phosphoryl chloride to yield the aldehyde,^20^ which was subsequently converted to the alcohol *via* hydride reduction (Scheme 1).^21^ After bromination of the alcohol,^22^ two TPA units were tethered through triazinanone under basic conditions. Deprotection of the urea afforded **3** as a pale yellow powder. Colorless needles were regularly obtained by slow evaporation of ethyl acetate solutions (∼20 mg mL^–1^) and were used for all solid-state measurements. The crystals were also subjected to X-ray diffraction analysis.

**Fig. 2:**
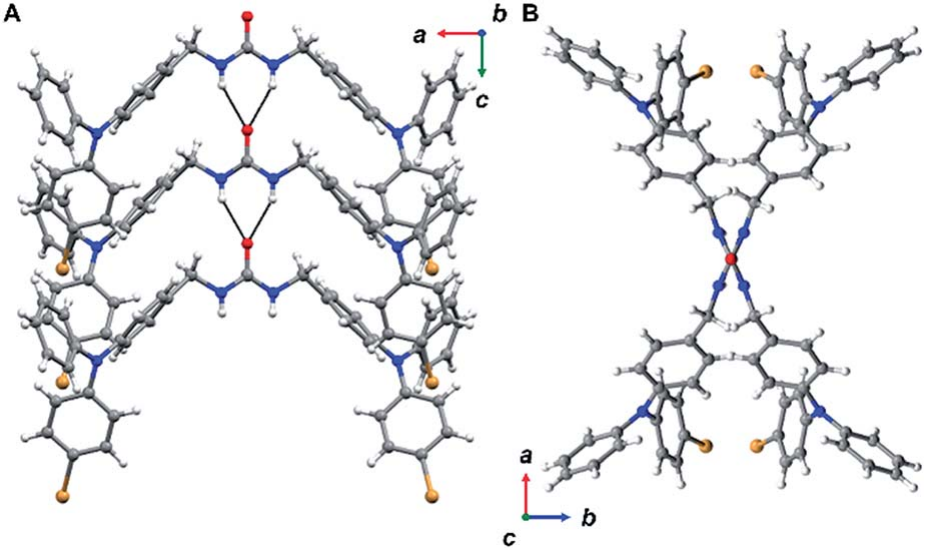
Views from **3** in the solid-state. Disorder was omitted for clarity. (A) Packing is driven through zig-zagged chains of urea hydrogen bonding. (B) Ureas adopt a twisting orientation creating an X-shape looking down the *c*-axis.

Triphenylamine **3** crystallized in the orthorhombic system in the *Pccn* space group. The X-ray structure revealed the desired compound with a linear *trans–trans* arrangement of the ureas with the two TPA units outstretched on both sides of the methylene urea tether in an anti-parallel manner. Crystallographically, the structure is disordered with two molecular orientations present (Fig. S9†) with the major component population of 91%. The urea carbonyl, which is located on a crystallographic *C*_2_ axis, is common to both components. The individual molecules are organized into chains extending along the crystallographic *c*-axis through characteristic three-centered urea hydrogen bonds with a twisting angle of 51.6(1)°. The hydrogen-bonded urea groups (N(H)···O distances of 2.823(3) and 2.70(2) Å, (Fig. 2A)) generate an X-shaped chain when viewed down the *c*-axis (Fig. 2B). The twisting is likely caused by the extra steric bulk of the TPA since similar dibenzylic systems typically have straight urea chains according to a Cambridge Structural Database survey (CSD 5.39, September 28, 2018).^23^

**Fig. 3:**
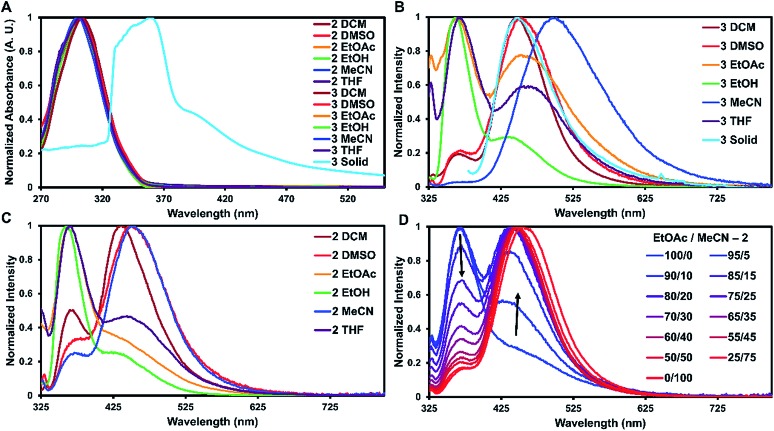
Absorbance and emission spectra of **2** and **3** at room temperature. (A) Normalized absorbance of **2** and **3** in different solvents and **3** in the solid-state. (B) Emission of **3** in different solvents and the solid-state. (C) Emission of **2** in different solvents. (D) Peak shift for **2** in the emission spectra changing from ethyl acetate to acetonitrile.

To examine how solvent and assembly affects the photophysics of the TPA compounds, the absorption and emission for **1**, **2**, and **3** were taken in six solvents and in the solid-state at room temperature. The studies in dichloromethane, dimethyl sulfoxide, ethyl acetate, ethanol, acetonitrile, and tetrahydrofuran are summarized in Table 1 and S2.† In all the tested solvents, the absorption spectra of **1–3** were nearly identical, with a strong ππ* transition at approximately 300 nm dominating the spectra with no other bands readily apparent (Fig. 3A). This suggests that in solution the proximity of the two TPA units have little effect on the absorption properties. On average, **2** and **3** were red-shifted by 2 nm compared to **1**. As expected, the molar absorptivity for **2** and **3** were very similar and twice that of **1** with values ranging from 4.70–5.53 × 10^4^ M^–1^ cm^–1^ and 2.12–2.68 × 10^4^ M^–1^ cm^–1^, respectively.

**Table 1:** Measured photophysical properties for compounds **2** and **3** under different conditions

Compound	Solvent	*λ*_abs_ ^*a*^ (nm)	*ε* (×10^4^ M^–1^ × cm^–1^)	*λ*_ems_ ^*b*^ (nm)	*τ*_<avg>_ ^*c*^ (ns)
**2**	DCM	304	5.11	365, 435*	0.8
	DMSO	302	5.42	451	5.1
	EtOAc	301	5.34	365	0.1
	EtOH	301	4.95	361	0.1
	MeCN	300	4.70	371, 451*	3.0
	THF	302	5.47	366*, 444	<0.1
**3**	DCM	303	5.20	366, 449*	2.2
	DMSO	302	5.41	369, 452*	4.1
	EtOAc	301	5.53	364*, 453	0.1
	EtOH	300	5.45	362*, 437	0.1
	MeCN	300	5.01	497	2.8
	THF	302	5.22	367*, 457	<0.1
	Solid	358	—	447	1.0

^*a*^Peak position at largest absorption band.

^*b*^Peak positions at largest emission bands in nm (largest denoted with * if applicable, excited at *λ*_abs._).

^*c*^Average lifetime of largest emission peak.

For solid-state samples, crystals of **3** were first examined by PXRD to probe if the bulk crystalline material was similar in structure to the single crystal of **3**. Fig. S11† compares the experimentally observed PXRD pattern to the predicted powder pattern simulated from the SC-XRD data. Seen here is an excellent correlation, suggesting that the bulk material is single phase and similar in structure to the solved crystal structure. This indicates that the photophysical measurements of the bulk material would be representative of the single crystals. Self-assembly of **3** resulted in a red shift of the absorbance of about 60 nm with slight broadening of the main peak (Fig. 3A). A similar red shift has been reported before for other triphenylamine derivatives on the basis of J-aggregates;^24^ however, this is more common for planar dyes.^25^

The emission spectra recorded in solution for **2** and **3** exhibited two main transitions either at approximately 370 nm (Band 1) and/or 450 nm (Band 2). As seen in Fig. 3B and C, the intensities of these bands varied widely on the basis of solvent with **2** exhibiting more Band 1 character and **3** more Band 2. Band 1 is generally considered the fluorescence band for TPA systems.^13^ To identify Band 2, further experiments were carried out.

First, the emission of **1** was taken in different solvents to probe if Band 2 was derived from an ICT process. Although ICT typically requires a donor–π–acceptor system,^26^ the TPA units of **2** and **3** could rotate over to each other allowing the TPA units on either side of the urea tether to act as both the donor and acceptor in the ICT exchange without the need for a π-system intermediate. For this case, the TPAs would have to adopt acceptor characteristics since TPAs are typically only the donor in ICT systems.^27^ As seen in Fig. S15,† **1**, which is only a single TPA unit, also exhibited Band 2. This suggests that band 2 is not due to an ICT process.

Second, DOSY NMR studies were carried out on **3** to probe if Band 2 originated from an AIE process. Since AIE has been known to create new emissive bands,^28^ ureas are known aggregators,^29^ and AIE has been observed in TPA systems before^14^ it seems reasonable that Band 2 could be derived from this process. For DOSY NMR, aggregation is detected when the observed hydrodynamic radius is significantly higher than the radius of the monomer. DOSY studies were conducted on solutions of **3** in deuterated acetonitrile (1 mM and 100 μM). This solution was chosen since it displayed the most significant Band 2 character of all the trials. As seen in Fig. S18 and S19,† no aggregation was observed for **3** in acetonitrile since the observed hydrodynamic radius of approximately 8 Å for both solutions is only slightly higher than that calculated from the crystal structure monomer of 3 (∼6 Å). The slightly higher radius may be from solvation or a slight amount of dimerization, but definitively no large-scale aggregation was observed. Considering that more dilute solutions (10 μM) were used for photophysical measurements and no aggregation was observed in more concentrated samples, this suggests that AIE is not responsible for Band 2.

With ICT and AIE ruled out for the occurrence of Band 2, phosphorescence is suggested as the likely origin. Phosphorescence is common for structures containing TPAs that are employed in OLED materials.^30^ Additionally, the peak position in the emission spectra is in good agreement for where phosphorescence is typically observed in TPA compounds. The bromine substituent can increase spin-orbital coupling *via* the heavy atom effect, which gives access to the triplet state thus enhancing phosphorescence.^17^ This effect can occur with either the heavy atom being directly connected into in the π-system,^31^ or in close proximity.^32^ The former case could explain why all three compounds exhibit Band 2, while the latter case could explain the intensity of this band (**3** > **2** > **1**).^30^ The emission spectra were also measured in the presence of a triplet quencher (triethylamine) and in an oxygen-saturated solution of dichloromethane. The emission was reduced in both cases (Fig. S31 and S32†), further suggesting that this band arises from phosphorescence.

For **1–3**, increasing solvent polarity resulted in increased phosphorescence, except in the case of polar protic solvents (ethanol) which showed little to no phosphorescence (Fig. 3B and C). Solvent dependent phosphorescence has been observed before when *S*_1_ and *T*_n_ were similar in energy.^33^ In this situation, different solvents stabilized either state in varying degrees resulting in different phosphorescent quantum yields for each solvent, which may be the case here as well. To further investigate the solvent dependence, we examined if phosphoresce could be ‘turned on’ by the addition of a polar solvent to a non-polar system (Fig. 3D). Starting with a fluorescent non-polar system (**2** in EtOAc), the addition of acetonitrile slowly turned on phosphorescence until the system was mostly phosphorescence clearly showing the solvent dependent nature of these emissive bands.

The luminescent lifetimes for **2** and **3** were found to be quite short for TPA derivatives with fluorescence and phosphorescent lifetimes estimated to be around 0.1 ns and 3 ns, respectively (Table 1, Fig. S20 and S21†). This may be due to competing non-radiative decay processes introduced by the heavy atoms. Typically, fluorescent lifetimes for TPA containing compounds tend to hover around 2 ns,^34^ but heavy atom containing TPA derivatives with small π-systems have be seen to exhibit shorter lifetimes (<0.1 ns).^22^ For the phosphorescent lifetimes, while the heavy atom effect does increase phosphorescence intensity^17^ it can shorten the lifetimes as well.^35^

In the solid-state it was not indicatively clear if fluorescence or phosphorescence was occurring as both the Stoke's shift (89 nm) and lifetime (1.0 ns) were in-between the expected values for fluorescence (65 nm, 0.1 ns) and phosphorescence (150 nm, 2.0 ns) determined from solution studies. Additionally, attempts at measuring accurate quantum yields for these compounds were unsuccessful due to radical generation and its subsequent effects on the photophysical properties.

The short observed lifetimes could be explained by the formation of stable radicals or by other non-radiative pathways. Thus, we turned to X-band EPR spectroscopy to probe radical formation within solution samples. First, a solution of **3** (∼1 mM) was prepared in degassed DCM and was sealed under argon. While no EPR signal was observed pre UV, UV-irradiation (1 h) of the sample resulted in an EPR signal with a *g*-value of 2.005 (Fig. S22†). However, this radical was unstable and found to rapidly undergo degradation reactions. Fig. S26† compares the ^1^H NMR spectra of **3** in solution before and after UV-irradiation. The post UV sample shows nearly a complete loss of all of the parent resonances. Post-UV absorption and emission spectra also followed this trend (Fig. S14 and S17†) clearly indicating that radicals of **3** generated in solution are not stable. This is not unexpected since electrochemical studies of control **1** indicate an unstable radical cation in solution^36^ and radical cations generated from TPAs with unsubstituted *para* positions are known to be unstable in solution.^9^,^10^

Next, EPR spectra were recorded on crystals of **3** in order to investigate how solid-state assembly influences the formation of radicals. First, EPR spectroscopy was performed on a triply recrystallized sample of **3** (3.9 mg) which was UV-irradiated for 6 h. Fig. S23† shows the recorded EPR spectra, which displays a broad signal with an axial powder pattern shape. The observed *g*-value is 2.006, which is in the range of TPA radical cations in solution (2.002–2.005).^5^ Singly recrystallized samples of **3** (10 mg) were also examined pre and post UV-irradiation (Fig. 1C). As expected, no signal was seen pre irradiation; however, after 1 hour of UV-irradiation a broad EPR signal identical to the triply recrystallized sample was observed.

**Fig. 4:**
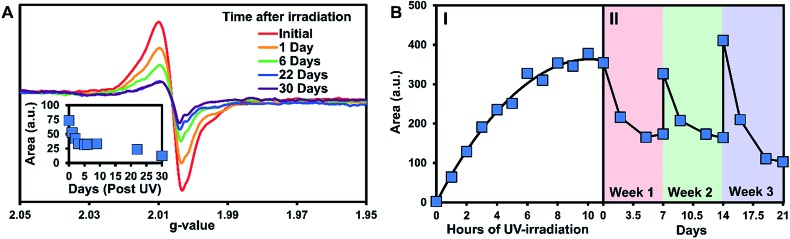
EPR data for **3** in the solid-state. (A) Dark decay after 1 hour of UV irradiation. Inset: the double integration of the dark decay spectra plotted *versus* time post UV irradiation. (B) (I) The double integration of the EPR spectra over time of UV irradiation followed by (II) a regeneration/decay study of the radicals. For part II, after the initial irradiation to the maximum radical concentration, the sample was irradiated for an additional 6 hours at the start of weeks 2 and 3.

The persistence of the photogenerated radicals was examined using dark decay studies in which the recrystallized sample was irradiated for one hour and then stored in the dark at room temperature under argon. The EPR spectrum was monitored over a month to estimate its stability (Fig. 4A). EPR signals were doubly integrated to obtain the area, which was plotted *versus* time after UV-irradiation (Fig. 4A, inset). A reliable radical signal persists up to a month with a half-life of approximately one week. After two months, when no radical signal was observable, we took a ^1^H NMR of the sample to see if the sample had degraded similarly to the solution study. Remarkably, the NMRs were identical to the initially synthesized materials indicating that **3** is photostable in its crystal form (Fig. S27†).

Next, we estimated the maximum concentration of radicals that could be generated through UV-irradiation by plotting the area of the EPR signal *versus* time exposed to UV light. The amount of radicals increases steadily with irradiation time (1 to 6 h) as seen in Fig. 4B part I. The plot of the double integration of the EPR signal *versus* time starts to plateau after 7–11 h of UV-irradiation with the crystals of **3** turning deep brown in color during the process (Fig. 1B). The concentration of radicals was approximated using a calibration with standard solutions of Magic Blue in DCM. Comparing the area of the EPR spectra of the solid-sample *versus* the Magic Blue calibration can give an approximate concentration of radicals generated in the solid-state. After 11 hours of UV-exposure, 9.0 mg of **3** generated the same amount of radicals as 100 μL of a 0.82 mM solution of Magic Blue, suggesting that 1 in 150 molecules of **3** have a radical (or 1 in 300 TPA units, Fig. S25†). Similar calculations for the 3.9 mg of the triply recrystallized sample were of a similar magnitude with ∼1 in 250 molecules exhibiting a radical after only 6 h of irradiation.

With no noticeable degradation of **3** occurring after radical formation in crystalline samples (Fig. S27†), we investigated if the radicals could be ‘regenerated’ after decay with repeated UV exposure. Typically, with chemical or electrochemical oxidations of TPAs to their corresponding radical cations, loss of the radical signal likely means the sample has degraded, and samples must be resynthesized. Remarkably, once the signal of **3** decays to half signal, irradiation with UV-light restores the radical concentration back to its maximum value (Fig. 4B, Part II). The samples were re-irradiated for 6 hours at the start of weeks 2 and 3 to regenerate the signal. As seen in Fig. 4B part II, the radicals decay at approximately the same rate over the three week long cycles. Also notable is that similar quantities of radicals are generated each time the crystals of **3** are UV-irradiated, demonstrating the exceptional stability and reproducible nature of the assembled structure *versus* in solution.

Next, we probed how the photogenerated radicals influenced the properties of the crystals as a whole. First, we compared the photophysics of the crystals before and after UV-irradiation (4 h). Both the absorption and emission were significantly quenched upon radical formation (Fig. S13 and S16†). This was quite striking considering the radical concentration was relatively low compared to the bulk sample. Oxidation of TPAs to radical cations is known to quench the photophysical properties,^8^ but typically it is quantitative in nature, at least in solution. This indicates that the generated radical is strongly delocalized to effect the whole system and behaves similarly to a radical cation.

To further probe the nature of the radical, irradiated (4 h) brown crystals of **3** were subjected to SC-XRD and IR spectroscopy. No change was observed in the overall single crystal structure; however, we noticed a minor change in the amount of disorder in the crystal going from 91% major conformer to 95%. This is likely correlated to the specific single crystal chosen for SC-XRD and is probably unrelated to radical formation. We expect that the TPA units are too bulky to self-correct during crystal formation leading to an array of different conformer percentages depending on the single crystal. For the IR studies, pre and post UV spectra were found to be nearly identical with no visible changes (Fig. S28†). The combination of SC-XRD and IR suggest that either the radical is highly delocalized and/or is not concentrated enough to be characterized by these methods.

**Fig. 5:**
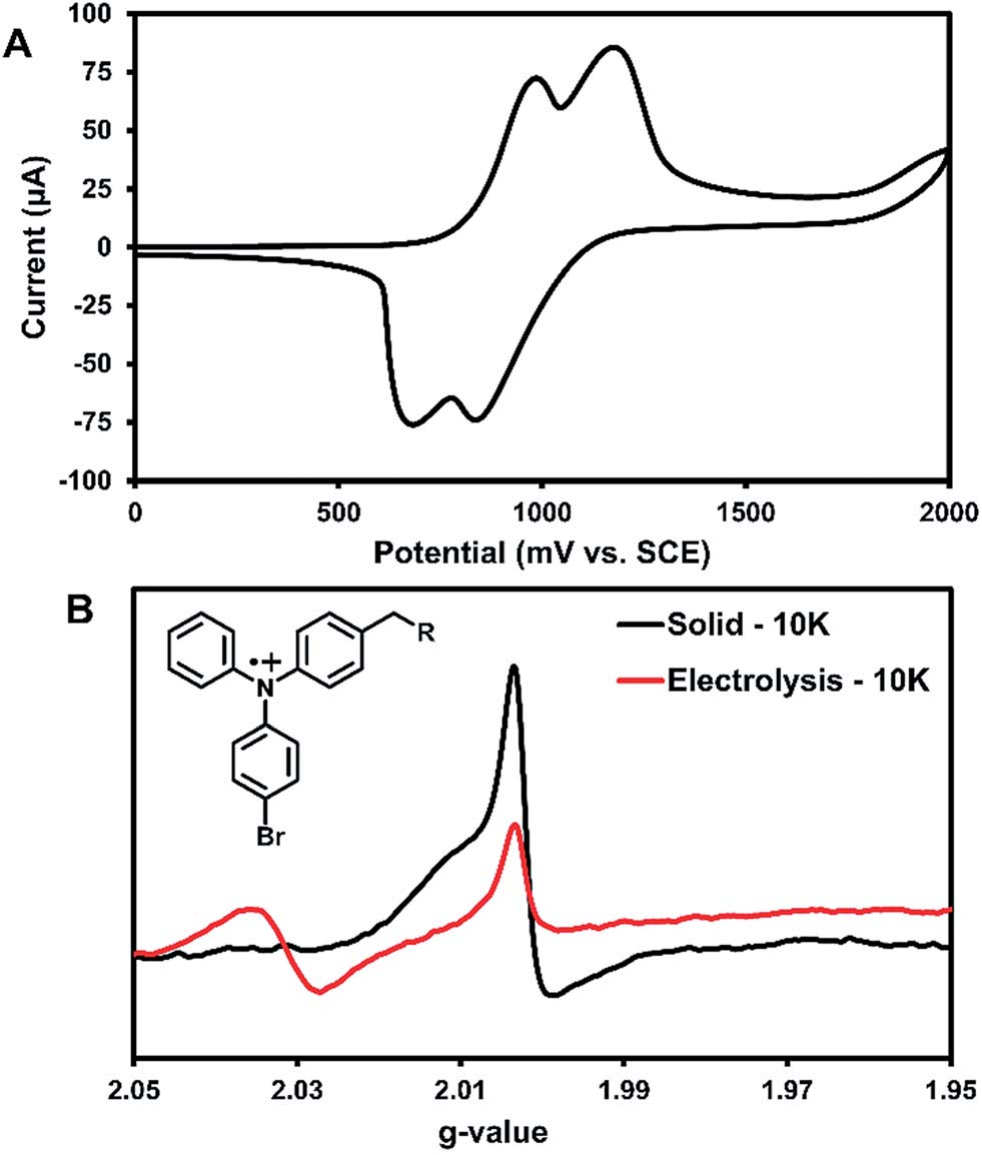
(A) CV for 1 mM **3** in a 0.1 M (*n*-Bu)_4_N^+^PF_6_^–^ DCM solution, scan rate 100 mV s^–1^. SCE = saturated calomel electrode. (B) Solution EPR for **3** at 10 K after bulk electrolysis at first oxidative peak (red) along with a solid-state EPR for **3** also at 10 K EPR after 3 hours of UV irradiation (black). Inset: proposed structure of radical responsible for EPR signal.

Cyclic voltammetry (CV) was used to further characterize the electronic structure of **3** in solution. Since the radicals in the solid-state acted similarly to TPA radical cations, this method could further characterize the types of radicals generated in this system. The oxidation of **3** in DCM shows two pseudo-reversible oxidation waves at 1.0 V and 1.2 V *vs.* SCE (Fig. 5A). In comparison, the oxidation of parent compound **1** leads to a degradation that was immediately visible in the CV,^36^ which suggests that **3** is more stable towards oxidation than the parent compound. Controlled potential electrolysis performed on **3** at +1.2 V passed at total of 3.98 electrons per molecule (Fig. S30†). Thus, each oxidative wave in Fig. 5A is attributed to a 2e^–^ oxidation of the symmetric compound **3**. Each electron is expected to come from the TPA units of the molecule, which would be consistent with previously reported results that have only one TPA unit per molecule.^36^ For comparison, an electrochemical study on compound **2** showed that slower scan rates (40 mV s^–1^) were required to obtain pseudo-reversible oxidation waves also near 1.0 V and 1.2 V *vs.* SCE (Fig. S29†). This indicates the rigidity of backbone of **2** has a clear impact on electron transfer kinetics.

Bulk electrolysis on a ∼1 mM solution of **3** in DCM was performed at the first oxidation peak to generate a radical cation in solution. The first peak was chosen for the oxidation since it likely affords an overall dication with each TPA unit being oxidized once. Electrolysis was performed for ∼5 hours to afford a bright yellow solution; however, once electrolysis was completed the resulting sample was unstable at room temperature and turned teal within 15 min. EPR analysis showed no signal. Thus, the electrolysis was performed on a new sample that was immediately immersed in liquid nitrogen for transportation and the EPR recorded at 10 K. As seen in Fig. 5B, two peaks for the electrolytic sample can be seen at *g*-values of 2.031 and 2.002.

The UV-generated radicals of crystalline **3** were also recorded at 10 K for comparison and showed no change in the line width and a slight shift in *g*-value to 2.002 compared to its room temperature spectrum, consistent with population of lower energy states. The peak at *g*-value = 2.002 was consistent in the electrolytic solution sample and the UV-irradiated crystals **3**. This suggests that crystalline **3** may form a similar radical species to electrolytic sample. Additionally, this is in good agreement with where TPA radical cations typically appear in EPR spectra.^5^ Thus, it is likely that the photogenerated radicals in assembled **3** are similar to radical triphenylamine cations formed by electrolysis (at *g*-value = 2.002), although it is not clear what anion is being formed in the crystalline sample for this process to occur. We are currently examining macrocyclic derivatives and are planning high field EPR studies that could help probe this question. The second isotropic signal at *g*-value = 2.031 was exclusive to the electrolyte sample and is attributed to degradation products.

Self-assembly of TPA urea dimers can stabilize the UV generated organic radicals in stark contrast to their solution counterparts. The concentration of the radicals is readily controlled by irradiation time up to a maximum of 1 in ∼150 molecules. The presence of the radicals can be visualized simply through their photophysical quenching behavior. Advantageous to this system is that the radicals can be generated in the solid-state without noticeable degradation to the starting materials and display a half-life up to a week. Additionally, these radicals can be regenerated upon re-irradiation without any loss in radical concentration. A comprehensive study on how different halogen substituents on these TPA compounds influences the radical generation, stability, and concentrations may be invaluable in revealing the factors that govern the photophysics of these compounds.

## Conclusions

In summary, a TPA methylene urea-tethered dimer was synthesized and readily afforded single crystals that organized the TPA through urea hydrogen bonding interactions. This solid-state assembly significantly stabilizes UV-generated radicals. Radicals formed in solution were unstable, as expected for incomplete *para* substituted TPA systems. In the solid-state, high quantities of radicals were formed, up to 1 in ∼150 molecules, which were persistent at room temperature with no observable degradation or significant changes in the single crystal X-ray diffraction. Further, radicals generated within the assembled framework have been shown to last up to a month with a half-life around a week. Most remarkably, after radical decay, radicals can be regenerated to their original maximum concentration with re-exposure to UV light. The photophysics of these materials were significantly quenched likely due to TPA hole transport properties even with relatively low radical concentration. Electrochemical evidence demonstrates that these compounds can be oxidized in solution at 1.0 V *vs.* SCE to generate radical cations, whose EPR spectra are similar to the UV-generated radicals in the solid-state. This suggests that the TPA radical cation is being formed in the solid-state and this electron transfer is reversible and reforms the parent compound over time. We are currently planning to carry out high-field EPR experiments as well as Dynamic Nuclear Polarization Magic Angle Spinning solid-state C13-NMR to further examine this process. Future work includes the synthesis of additional halogenated on the TPA analogs to elucidate the factors that govern radical formation, persistence, and quantity. Understanding how assembly enhances the stability of radicals would be exceedingly helpful in the end goal of making better conductive and magnetic materials that incorporate TPA scaffolds.

## Conflicts of interest

There are no conflicts to declare.

## Supplementary Material

Supplementary information

Crystal structure data
