# Supplementary material for: UV-irradiation of self-assembled triphenylamines affords persistent and regenerable radicals
Source: Chem Sci. 2019 Jan 10;10(9):2670–7. doi: 10.1039/c8sc04607g (PMC6419929; doi:10.1039/c8sc04607g)
Supplement: Supplementary file 1 [file SC-010-C8SC04607G-s001.pdf]

Electronic Supplementary Material (ESI) for Chemical Science.
This journal is © The Royal Society of Chemistry 2019

Supplementary Information for

UV-irradiation of self-assembled triphenylamines affords persistent and regenerable radicals

Ammon J. Sindt, Baillie A. DeHaven, David F. McEachern Jr., D. M. M. Mevan Dissanayake, Mark D. Smith, Aaron K. Vannucci, and Linda S. Shimizu*

Department of Chemistry and Biochemistry, University of South Carolina, Columbia, South Carolina 29208, United States

Fax: 803-777-9521; *Tel:* 803-777-2066

E-mail: SHIMIZLS@mailbox.sc.edu

Contents

General experimental	**2**
Synthesis and characterization of compounds	**3**
Crystal data and structure refinement	**16**
Powder X-ray diffraction	**21**
Absorbance measurements	**22**
Emission measurements	**24**
Photophysical values for **1**-**3**	**26**
DOSY NMR	**27**
Lifetime calculations and measurements	**29**
EPR measurements	**32**
NMR spectra pre and post UV	**35**
Infrared measurements	**37**
Cyclic voltammetry	**38**
Bulk Electrolysis	**39**
Emission Quenching	**40**
References	**42**

General Experimental

NMR spectra were recorded on Bruker Avance 300 or 400 MHz spectrometers. Chemical shifts are reported in ppm (δ) and were internally referenced with the solvent peak. All chemicals were purchased from chemical suppliers and were used as received unless otherwise noted. High-resolution mass spectrum data were recorded using a direct exposure probe (DEP) in electron ionization mode on a Waters QTOF-I quadrupole time-of-flight mass spectrometer. UV-irradiation of all materials were carried out with a Hanovia 450 W medium pressure mercury arc lamp cooled in a quartz immersion well. Samples were purged with argon before irradiation. All other instrument protocols are described in their own sections hereafter.

Synthesis and Characterization of Compounds

Scheme S1. Synthesis of compounds.

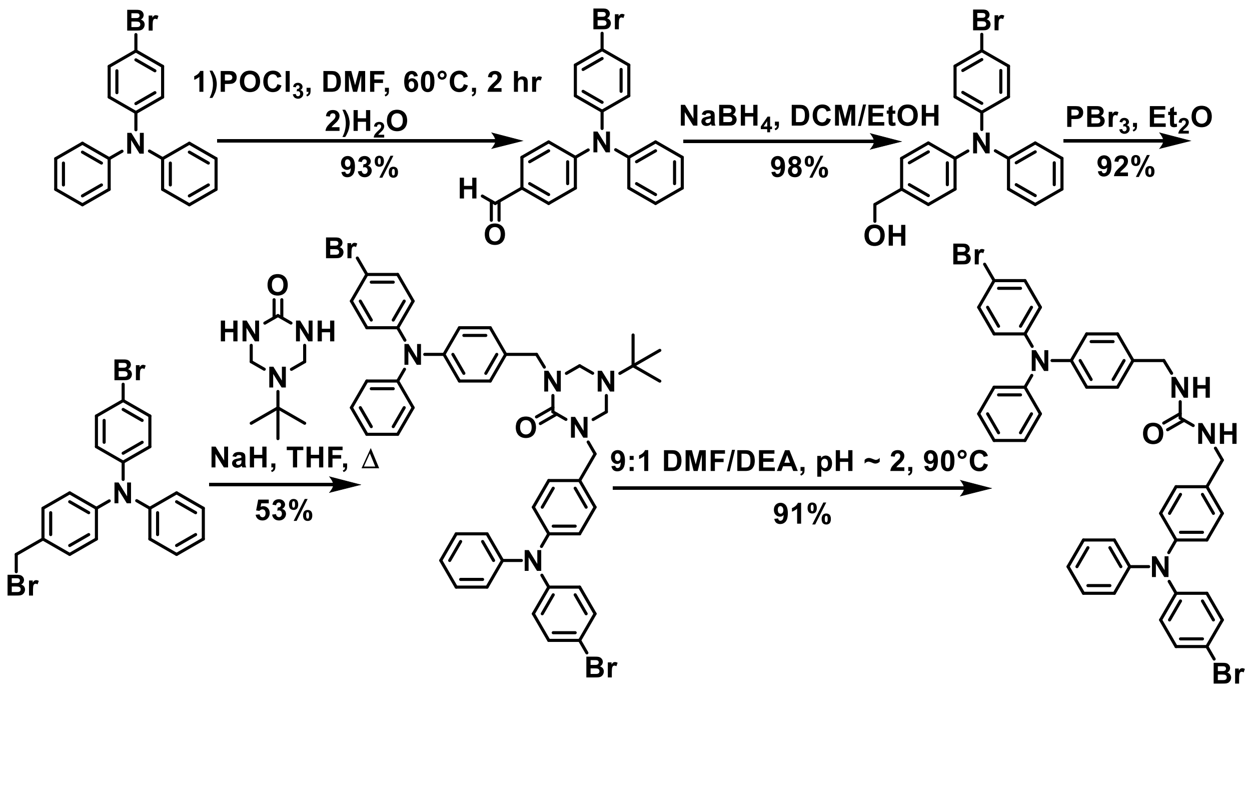

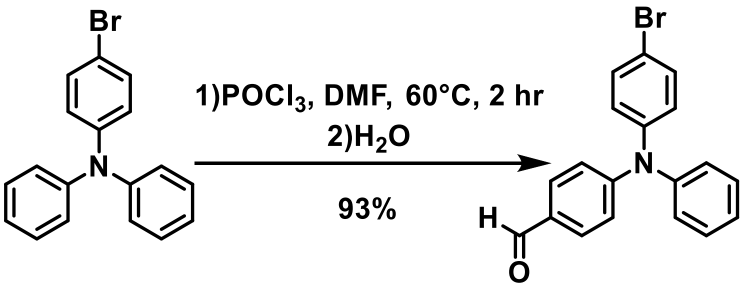

4-((4-Bromophenyl)(phenyl)amino)benzaldehyde: Compound was made according to previous procedures.[1] Phosphoryl chloride (840 μL, 9.0 mmol) was added dropwise to dry *N, N*-dimethylformamide (830 μL, 10.8 mmol) at 0˚C and the mixture was stirred at room temperature for 20 minutes. Then 4-bromo-*N, N*-diphenylaniline (2.590 g, 8.0 mmol) was added and this mixture was heated to 110˚ C then cooled to stir at 60˚ C for 2 hours. After cooling to room temperature, 50 mL of ice-cold water was added to the mixture and the solution was neutralized with saturated $NaHCO_{3(aq)}$. The mixture was extracted with chloroform (3 x 50 mL) and the organics were washed with water (1 x 50 mL), then brine (1 x 50 mL), and dried with $MgSO_4$. The solvent was removed via rotary evaporation and the crude material was further purified by column chromatography (Hexanes/Ethyl Acetate = 3:1) to yield the product as a yellow solid (93%). Spectra matched that as previously reported.[1] ^{1}H NMR (300 MHz, $CDCl_3$): δ (ppm) 9.82 (s, 1H), 7.69 (d, *J* = 8.5 Hz, 2H), 7.44 (t, *J* = 8.5 Hz, 2H), 7.35 (t, *J* = 7.8 Hz, 2H), 7.22-7.11 (m, 3H), 7.08-6.99 (m, 4H).

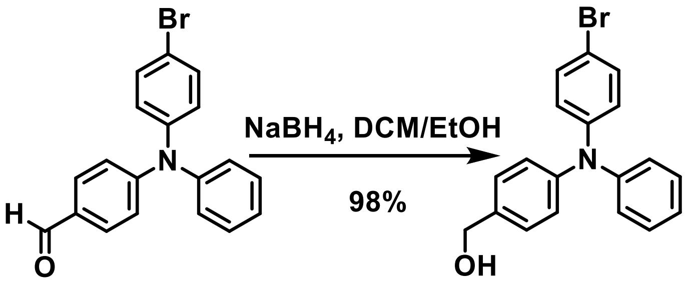

(4-((4-Bromophenyl)(phenyl)amino)phenyl)methanol: Compound was made according to previous procedures.[2] The previous aldehyde (2.609 g, 7.4 mmol) was dissolved in 120 mL of a 3:1 mixture of dry dichloromethane and ethanol. Sodium borohydride (0.308 g, 8.2 mmol) was added after and the mixture stirred at room temperature for 1 day in the dark. Then 180 mL of water was added and the mixture was extracted with dichloromethane (3 x 70 mL) and dried with $MgSO_4$. Solvent was removed via rotary evaporation leaving behind the alcohol as a sticky solid (98%). ^{1}H NMR (300 MHz, $(CD_3)_2SO$): δ (ppm) 7.41 (d, *J* = 8.8 Hz, 2H), 7.34-7.24 (m, 4H), 7.09-6.97 (m, 5H), 6.86 (d, *J* = 9.0 Hz, 2H), 5.14 (t, *J* = 5.8 Hz, 1H), 4.45 (d, *J* = 5.9 Hz, 2H). ^{13}C NMR (75 MHz, $CDCl_3$): δ (ppm) 147.42, 147.11, 147.07, 135.73, 132.32, 129.55, 128.51, 125.32, 124.59, 124.47, 123.49, 115.06, 65.18. HRMS (DEP): [M+] calculated, 353.0415; found, 353.0409.

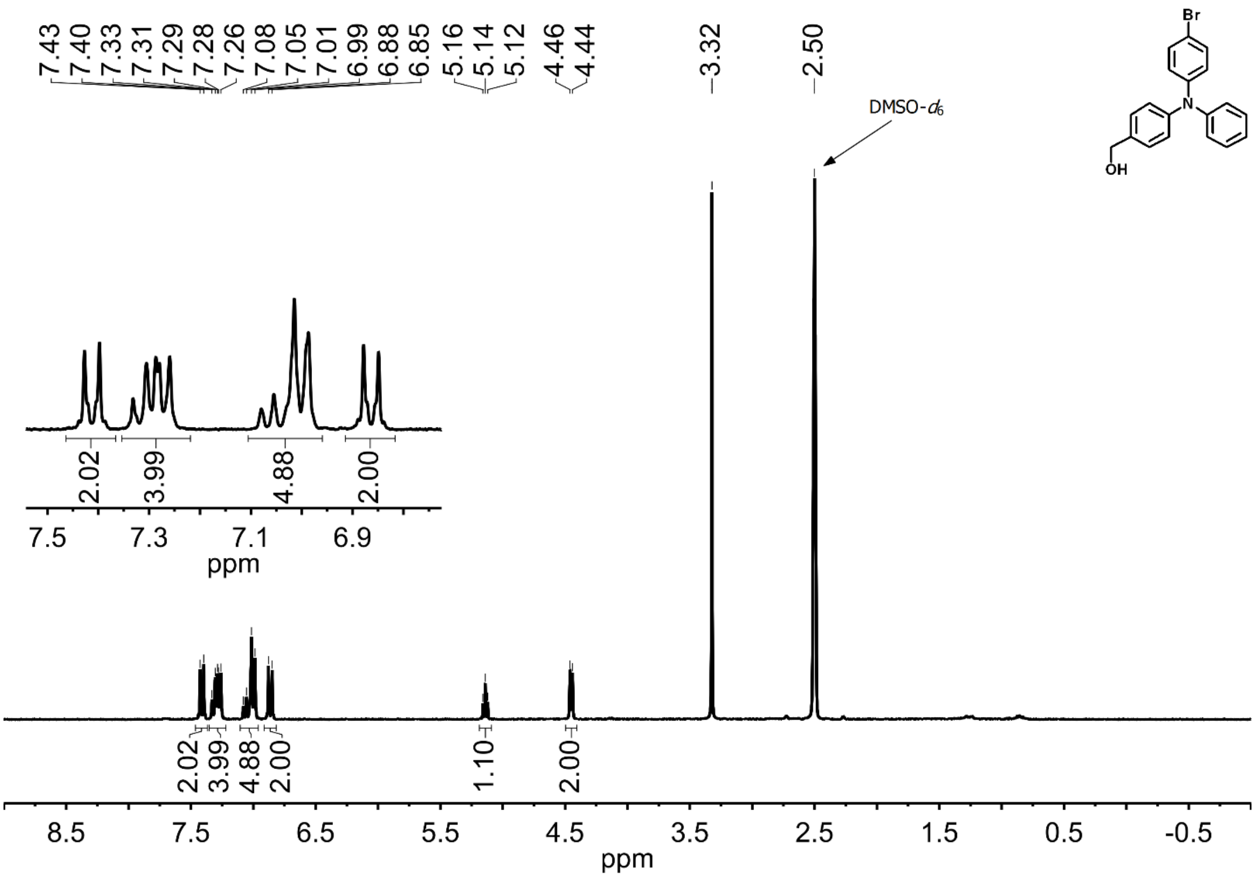

Figure S1. ^{1}H NMR ($(CD_3)_2SO$, 300 MHz)

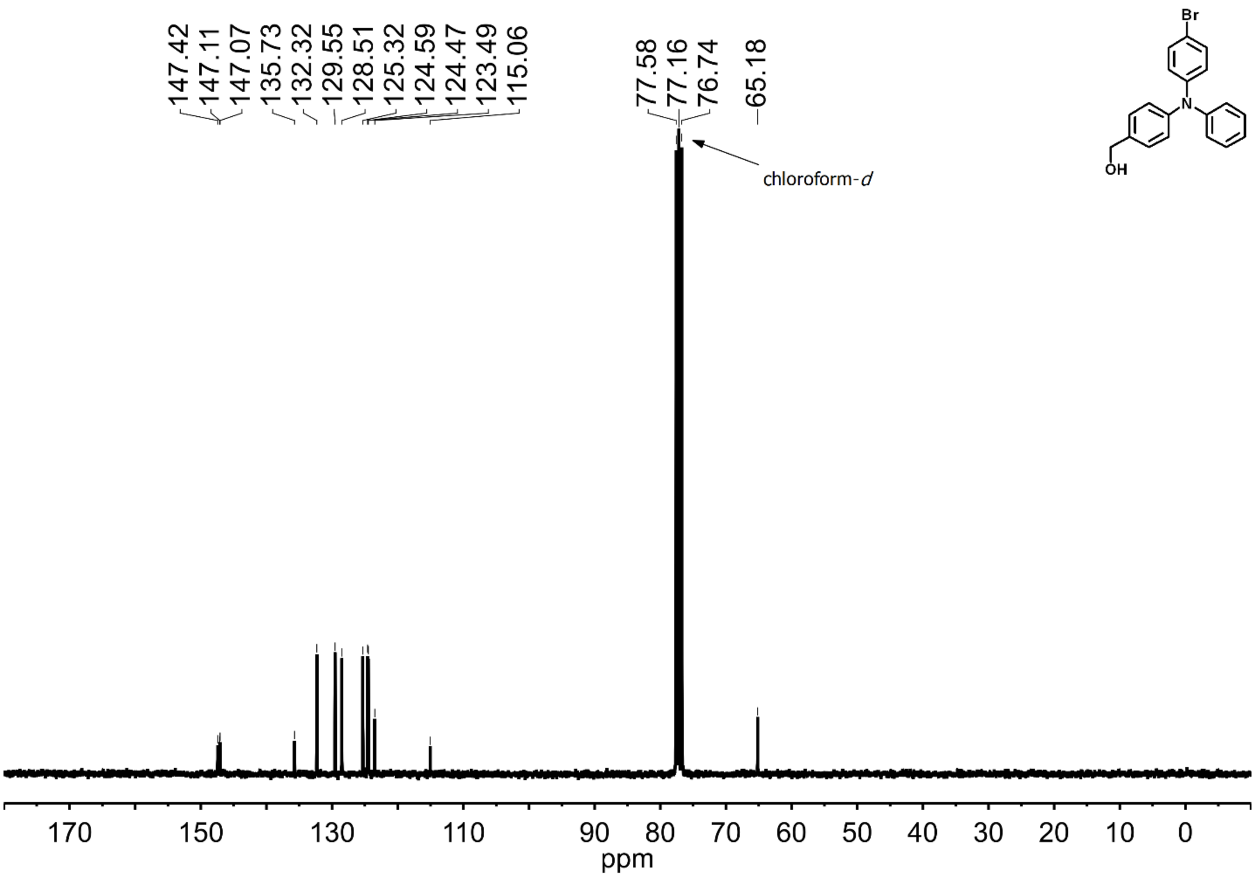

Figure S2. ^{13}C NMR ($CDCl_3$, 75 MHz)

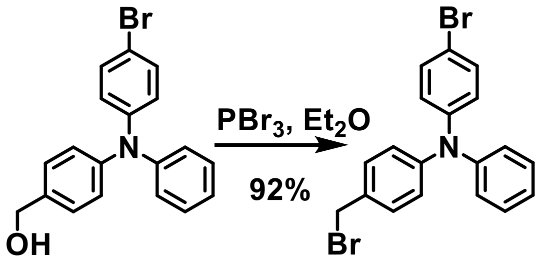

4-Bromo-*N*-(4-(bromomethyl)phenyl)-*N*-phenylaniline: Compound was made according to previous procedures.[3] The previous alcohol (2.253 g, 6.4 mmol) was suspended in 80 mL of dry diethyl ether and was cooled to 0 ˚C. Then a solution of phosphorus tribromide (363 µL, 3.8 mmol) in 10 mL dry diethyl ether was added dropwise over 5 minutes. The reaction stirred at room temperature overnight in the dark. In the morning, ice cold water (90 mL) and saturated $NaHCO_{3(aq)}$ (45 mL) was added to quench the reaction. The mixture was extracted with (1 x 90 mL) of dichloromethane and the organics were washed with brine (3 x 45 mL) and dried with $MgSO_4$. The solvent was removed under rotary evaporation to yield the bromide as a sticky solid (92%). ^{1}H NMR (300 MHz, CD_2Cl_2): δ (ppm) 7.40-7.23 (m, 6H), 7.13-7.04 (m, 3H), 7.03-6.92 (m, 4H), 4.51 (s, 2H). ^{13}C NMR (75 MHz, CD_2Cl_2): δ (ppm) 147.99, 147.36, 147.06, 132.63, 132.34, 130.58, 129.90, 126.09, 125.43, 124.27, 123.69, 115.69, 34.46. HRMS (DEP): [M+] calculated, 414.9571; found: 414.9580.

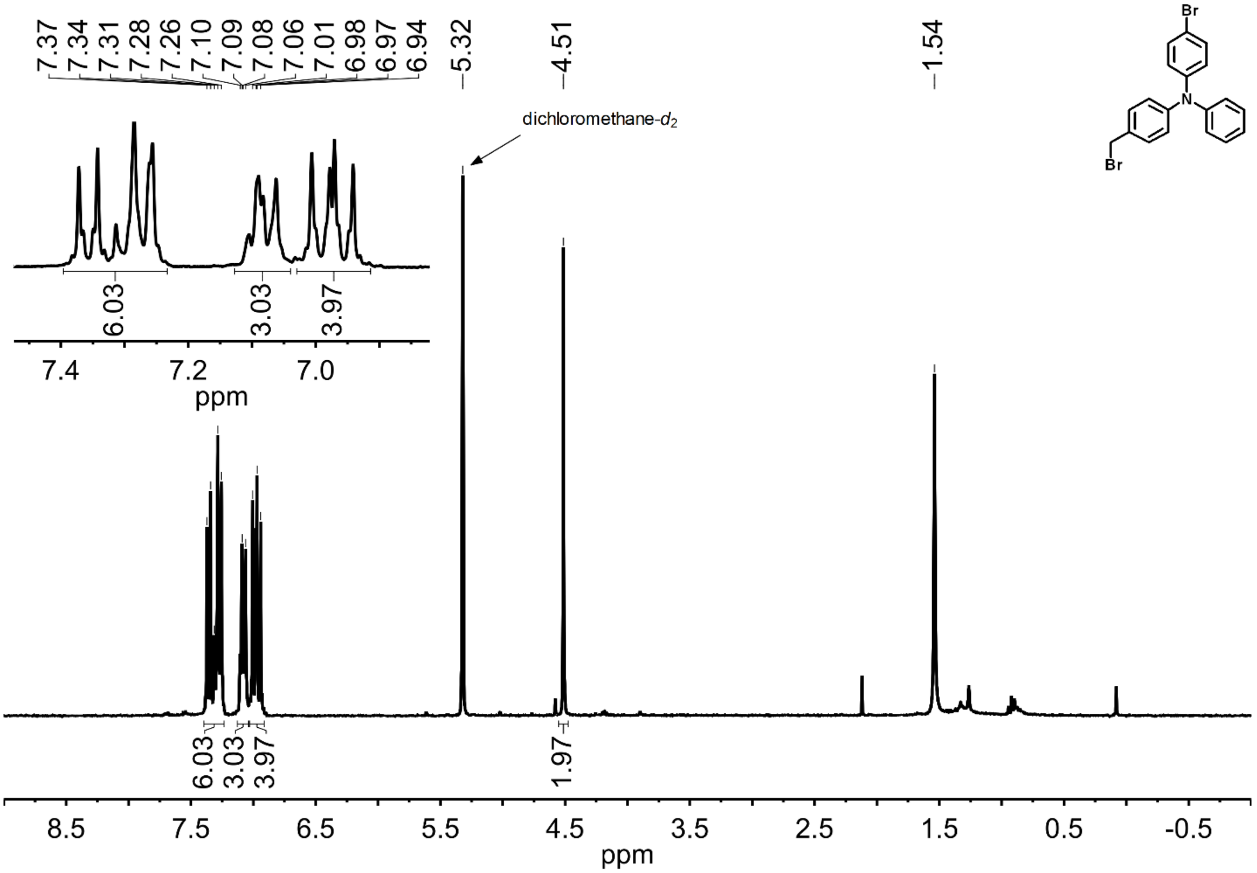

Figure S3. ^{1}H NMR (CD_2Cl_2, 300 MHz)

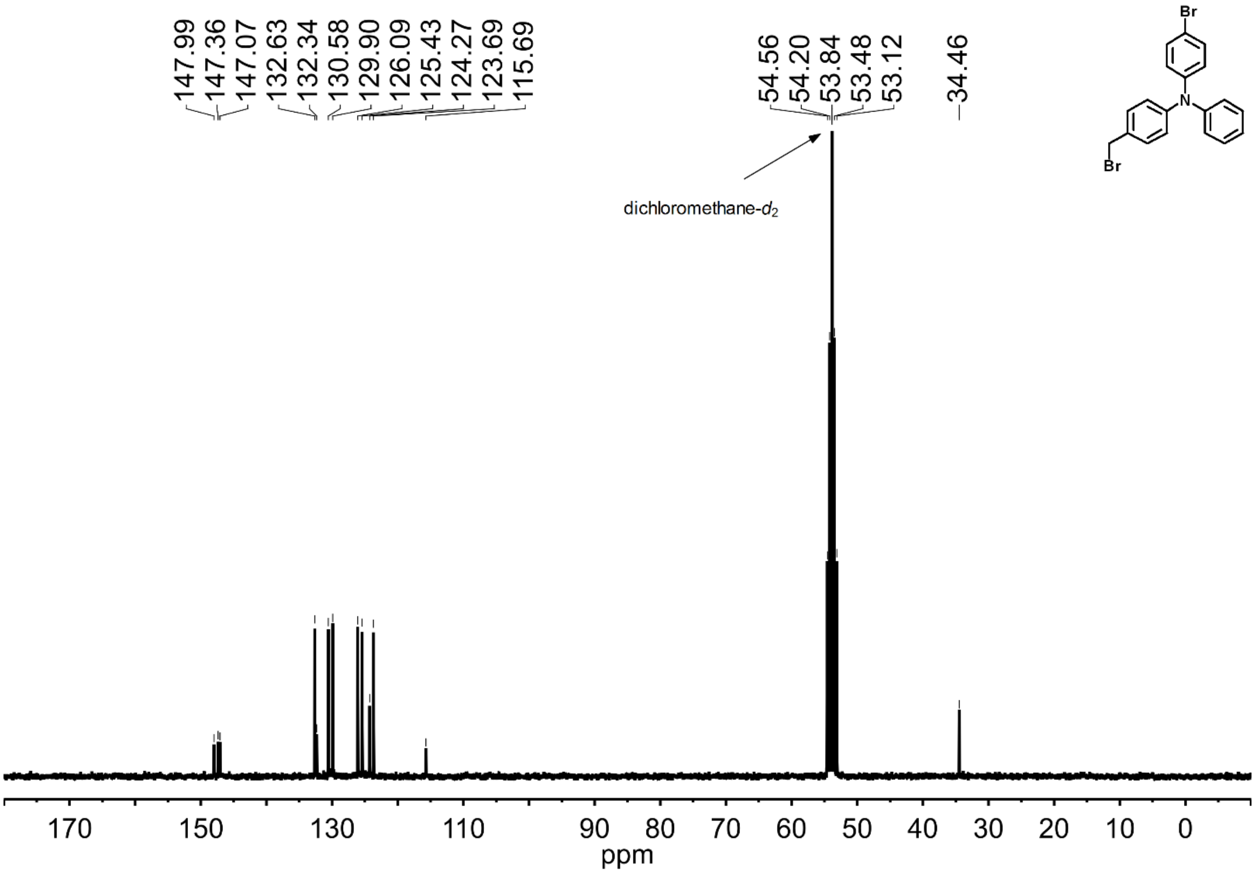

Figure S4. ^{13}C NMR (CD_2Cl_2, 75 MHz)

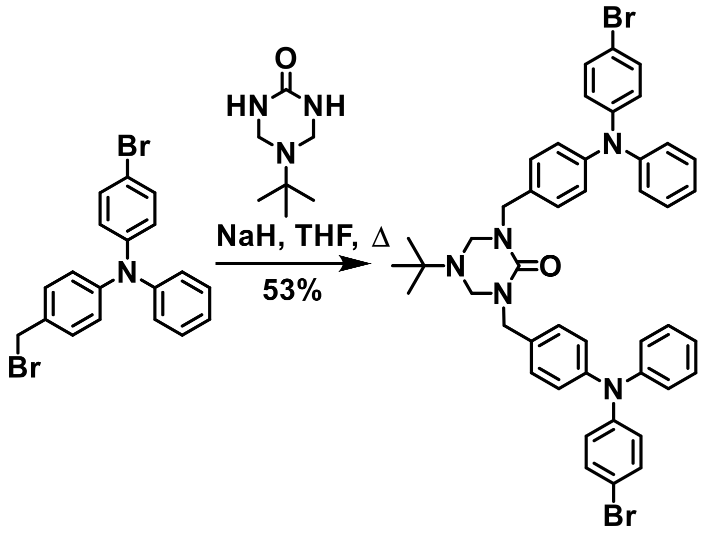

1,3-*bis*(4-((4-bromophenyl)(phenyl)amino)benzyl)-5-(*tert*-butyl)-1,3,5-triazinan-2-one: *tert*-Butyl triazinanone (0.209 g, 1.3 mmol) and sodium hydride (60% suspension in paraffin oil, 0.160 g, 4.0 mmol) were suspended in 20 mL of dry tetrahydrofuran and was stirred for 10 minutes. Then the previous bromide (1.111 g, 2.7 mmol) was added as a solution in 20 mL of dry tetrahydrofuran. The reaction stirred at reflux in the dark overnight. After cooling to room temperature, 6 mL of both 1 N $HCl_{(aq)}$ and water were added to quench the reaction. This solution was extracted with dichloromethane (3 x 60 mL). The organics were washed with brine (1 x 60 mL) and dried with $MgSO_4$. The solvents were removed under rotary evaporation, and the product was isolated using column chromatography (Hexanes/Ethyl Acetate = 2:1) to yield the product as a sticky solid (53%). ^{1}H NMR (300 MHz, $(CD_3)_2CO$): δ (ppm) 7.43-7.26 (m, 12H), 7.10-7.01 (m, 10H), 6.93 (d, *J* = 8.9 Hz, 4H), 4.51 (s, 4H), 4.34 (s, 4H), 1.02 (s, 9H). ^{13}C NMR (75 MHz, $(CD_3)_2CO$): δ (ppm) 156.50, 148.27, 148.24, 147.16, 135.49, 132.99, 130.63, 130.36, 125.63, 125.44, 125.16, 124.27, 114.80, 62.39, 54.83, 48.39, 28.74. HRMS (DEP): [M+] calculated, 828.1907; found, 828.1927.

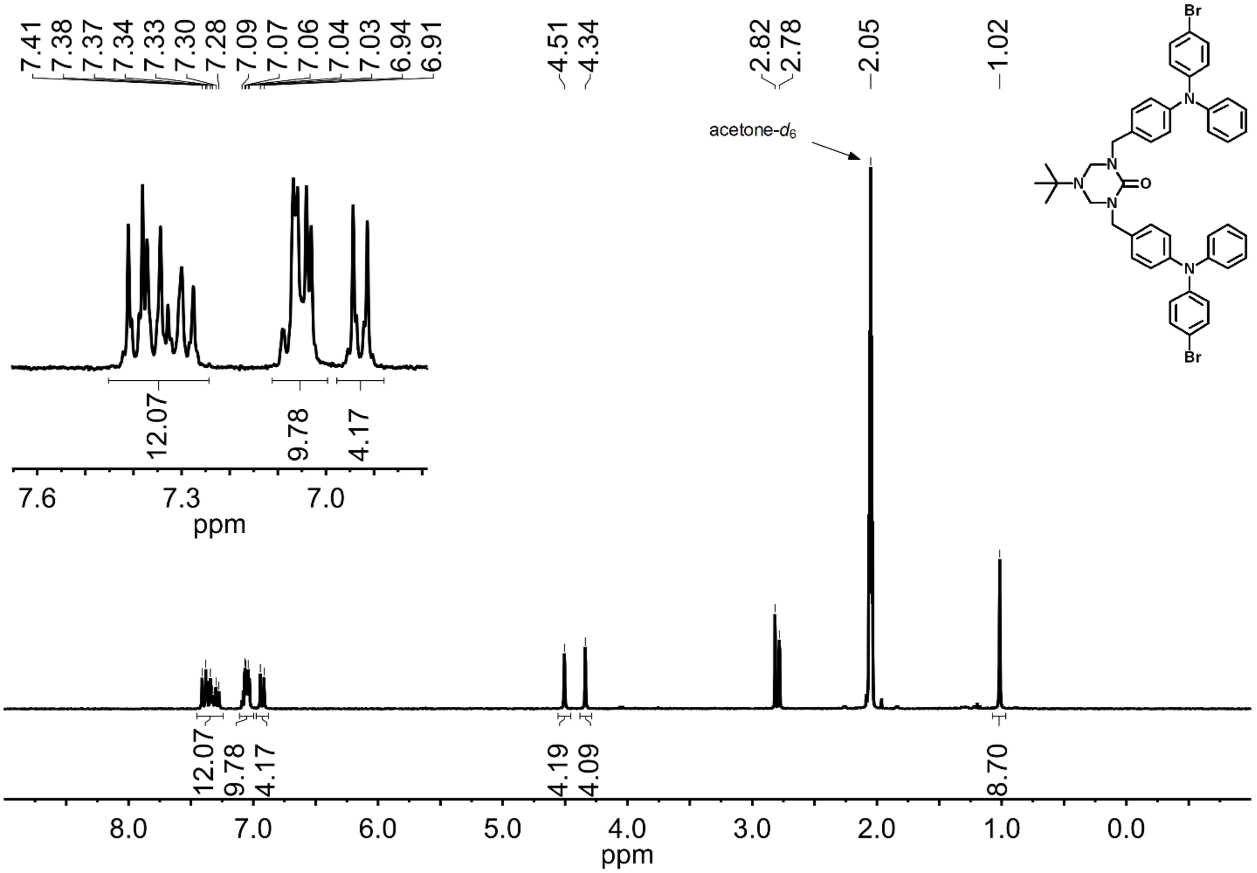

Figure S5. ^{1}H NMR ($(CD_3)_2CO$, 300 MHz)

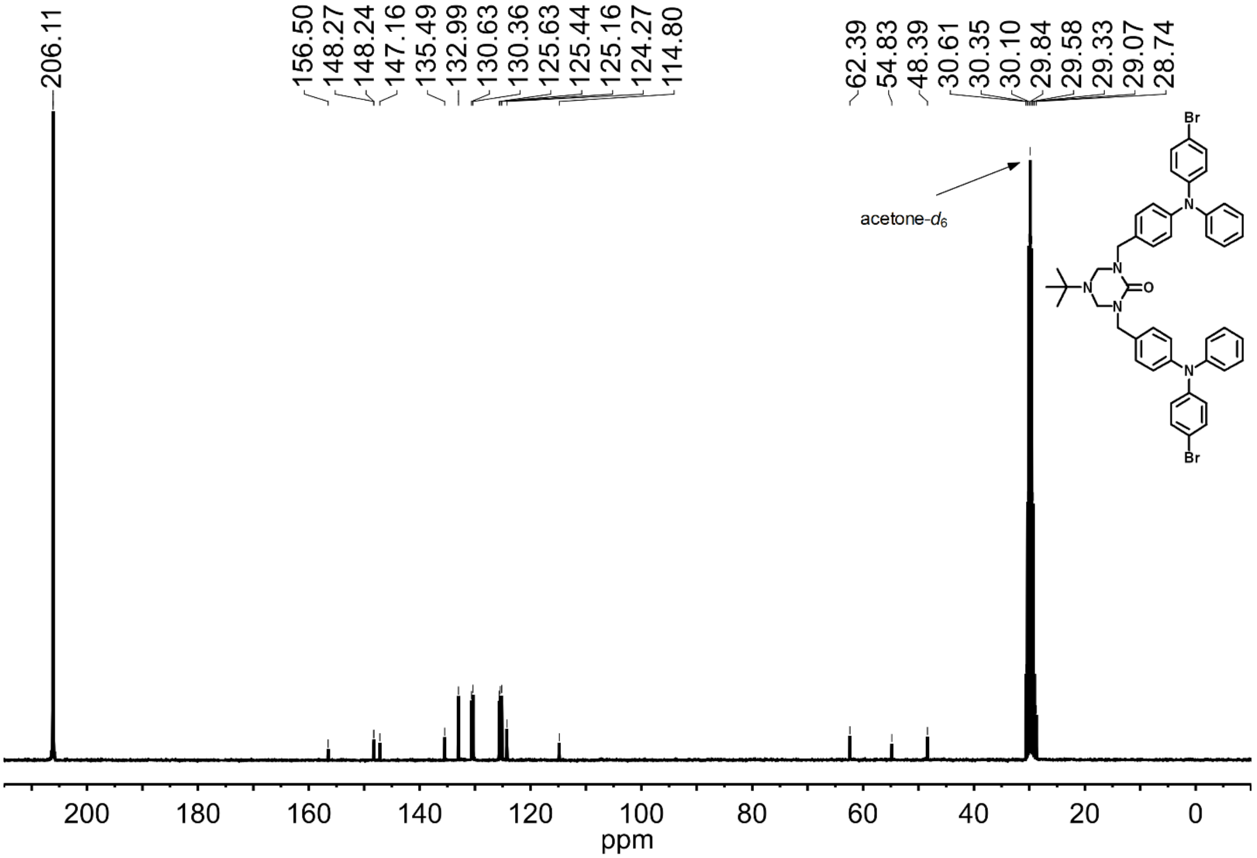

Figure S6. ^{13}C NMR ($(CD_3)_2CO$, 75 MHz)

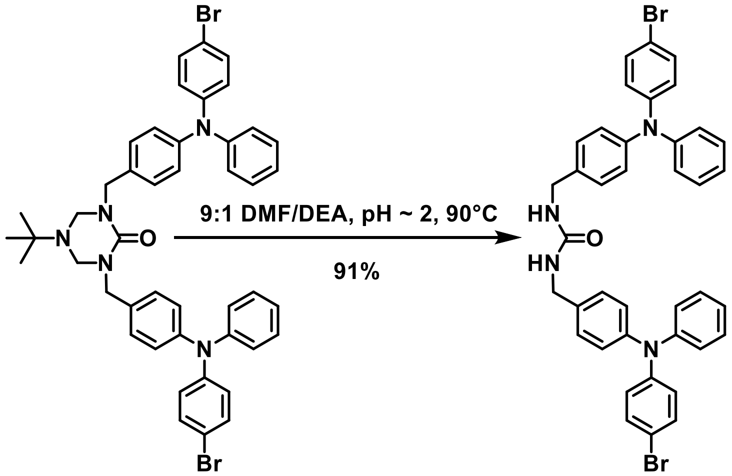

1,3-*bis*(4-((4-bromophenyl)(phenyl)amino)benzyl)urea: The previous protected urea (0.100 g, 0.1 mmol) was suspended in 50 mL of a 9:1 solution of *N, N*-dimethylformamide and diethanol amine and the pH was adjusted to 2 using 12 M $HCl_{(aq)}$. This mixture was heated at 90˚C for 2 days in the dark. The pH was readjusted to 2 using 12 M $HCl_{(aq)}$ every 12 hours until completion. After cooling to room temperature, the reaction was filtered and the residue was washed with 50 mL of water leaving behind the product as a beige solid (91%).^{1}H NMR (300 MHz, CD_2Cl_2): δ (ppm) 7.36-7.13 (m, 12H), 7.09-6.96 (m, 10H), 6.90 (d, 8.8 Hz, 4H), 4.70 (t, *J* = 5.8 Hz, 2H), 4.32 (d, *J* = 5.8 Hz, 4H). ^{13}C NMR (75 MHz, $CDCl_3$): δ (ppm) 158.04, 147.35, 147.00, 146.79, 133.81, 132.33, 129.56, 128.64, 125.27, 124.57, 124.47, 123.53, 115.07, 44.30. HRMS (DEP): [M+] calculated, 731.1016 found, 731.1025.

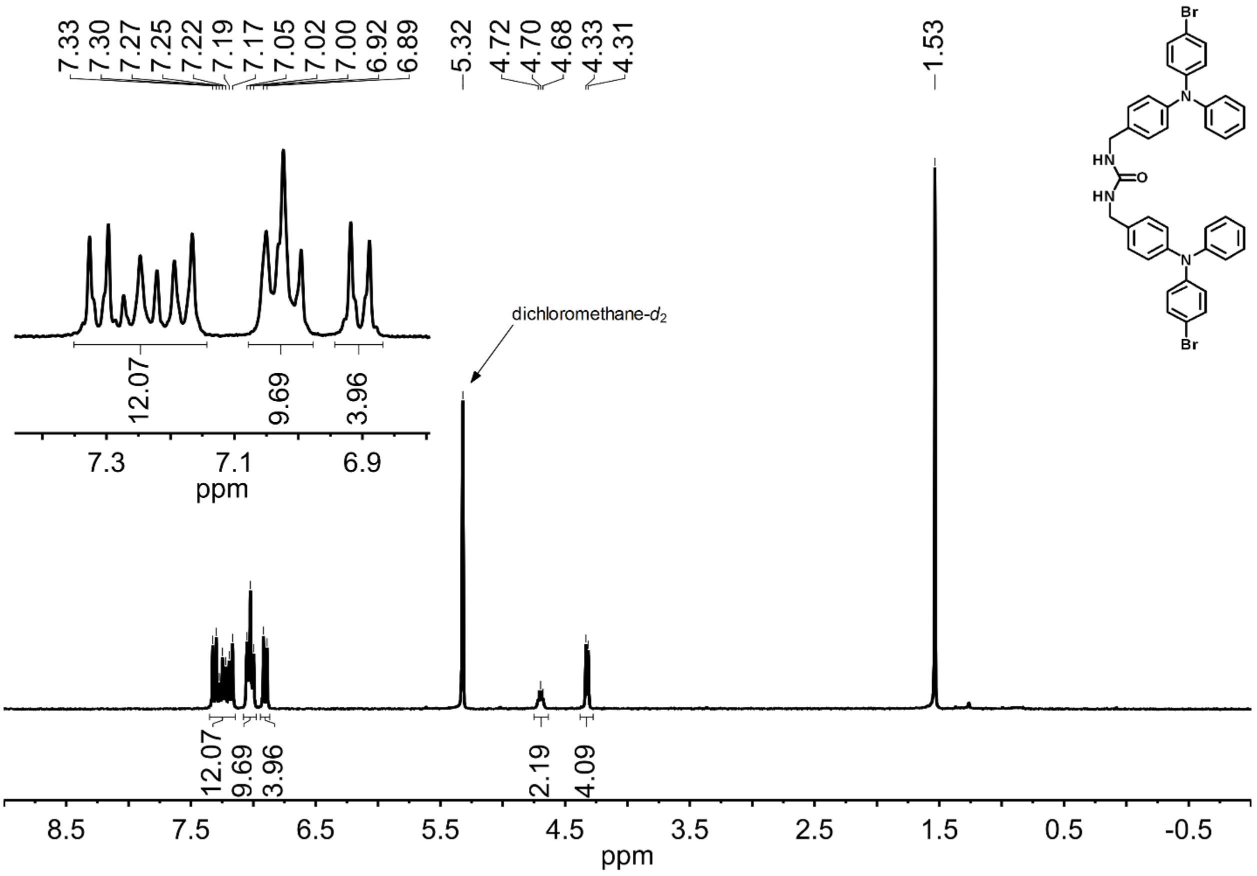

Figure S7. ^{1}H NMR (CD_2Cl_2, 300 MHz)

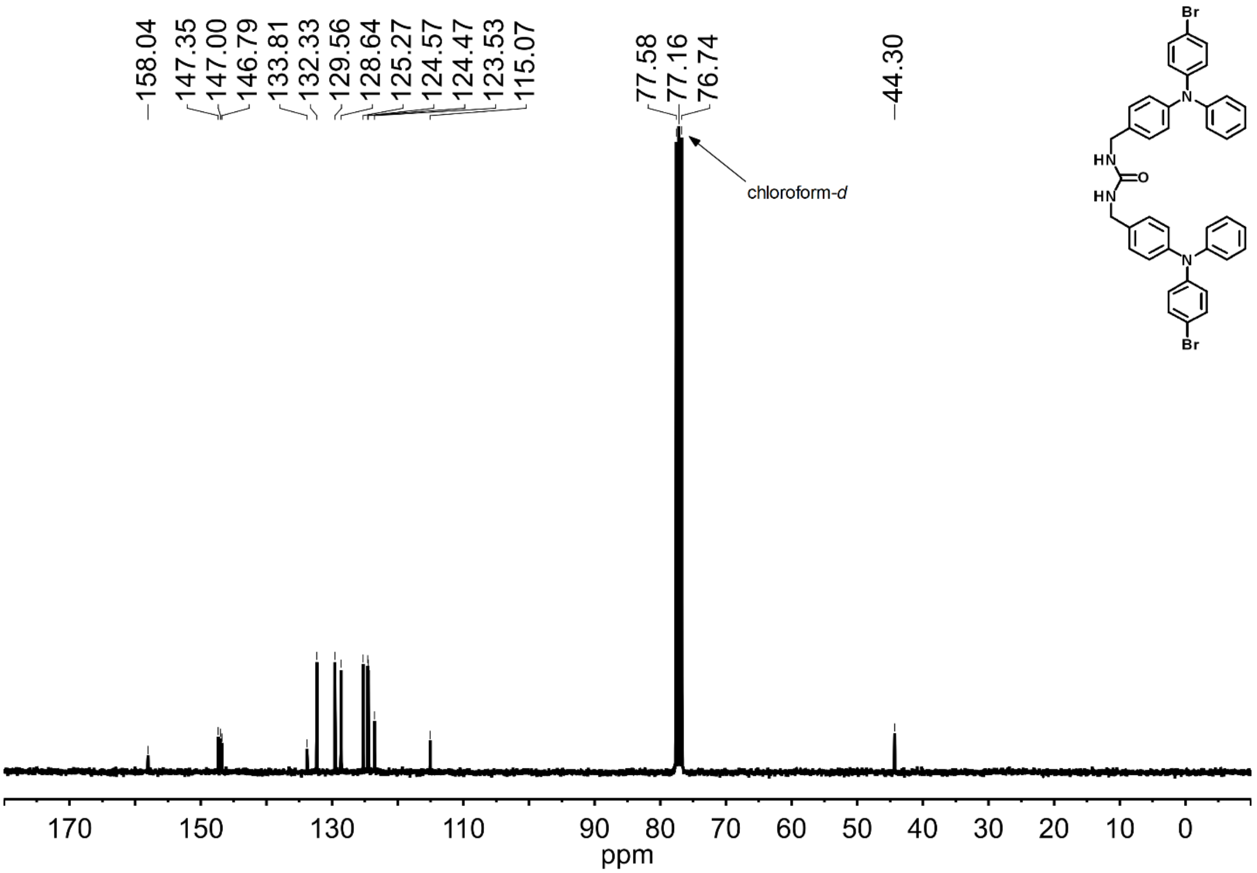

Figure S8. ^{13}C NMR ($CDCl_3$, 75 MHz)

Single Crystal X-ray Diffraction (SC-XRD)

X-ray intensity data from a colorless needle were collected at 100(2) K using a Bruker D8 QUEST diffractometer equipped with a PHOTON-100 CMOS area detector and an Incoatec microfocus source (Mo Kα radiation, λ = 0.71073 Å). The raw area detector data frames were reduced and corrected for absorption effects using the Bruker APEX3, SAINT+ and SADABS programs.[4,5] Final unit cell parameters were determined by least-squares refinement of 9851 reflections taken from the data set. The structure was solved with SHELXT.[6] Subsequent difference Fourier calculations and full-matrix least-squares refinement against F^2 were performed with SHELXL-2018[3] using OLEX2.[7]

The compound crystallizes in the orthorhombic system. The pattern of systematic absences in the intensity data was uniquely consistent with the space group *Pccn*, which was verified by structure solution. The asymmetric unit consists of half of one molecule, which is located on a two-fold axis of rotation. The structure is disordered. After routine location and anisotropic refinement of the unique half of the molecule, a very large residual electron density peak (magnitude 4.3 e-/Å3) was observed in a chemically implausible site, ca. 2.0 Å from Br1. After ruling out data processing errors (crystal slippage, poor integration parameters, absorption correction, e.g.) the peak was eventually interpreted as the bromine atom of a minor 'whole-molecule' disorder component. Refinement of the site occupancy of this peak assigned as bromine and the major component bromine atom site occupancy summed to near one, providing support for this model. Subsequently, other peaks were picked from difference Fourier maps to complete the unique half of the minor disorder component, also located on the two-fold axis. The carbonyl C and O atoms are common to both disorder components. 1,2- and 1,3-distances in the minor disorder component (atoms label suffixes "B") were restrained to be similar to those in the major component (atom label suffixes "A") via a SHELX SAME instruction. Anisotropic displacement parameters for atoms which are overlapped were held equal. An additional FLAT instruction was necessary for ring C9B-C14B, and Br1B was restrained to be equidistant from C11B and C13B. The

disorder fractions were constrained to sum to one, and refined to A/B = 0.910(1) / 0.090(1). The same large residual peak / whole molecule disorder was observed in two other crystals. No sign of the disorder was evident in the diffraction frames, and no resolving twin law was found from the TwinRotMat program.[8] The modeled disorder suggests a deviation from ideal C_2 point symmetry in approximately 9% of molecules throughout the crystal. Solution in lower space groups lacking imposed C_2 symmetry showed the same disorder. All non-hydrogen atoms were refined with anisotropic displacement parameters. Hydrogen atoms bonded to carbon were placed in geometrically idealized positions and included as riding atoms with d(C-H) = 0.95 Å and Uiso(H) = 1.2Ueq(C) for aromatic hydrogen atoms, and d(C-H) = 0.99 Å and Uiso(H) = 1.2Ueq(C) for methylene hydrogen atoms. One unique position for the hydrogen atoms bonded to the urea nitrogen was located. It was refined isotropically with distances to both N1A and N1B restrained to be 0.84(2) Å. The largest residual electron density peak in the final difference map is 0.86 e-/Å^3, located 0.67Å from H11B. Crystal data are given in Table S1.

Table S1. Data Collection and Refinement for Crystals

Identification Code	3
CCDC	1873066
Color of Crystal	Colorless
Empirical formula	$C_{39}H_{32}Br_2N_4O$
Formula weight	732.50
Temperature/K	100(2)
Crystal system	orthorhombic
Space group	Pccn
a/Å	18.5184(8)
b/Å	19.9443(7)
c/Å	9.0043(4)
α/deg	90
β/deg	90
γ/deg	90
Volume/Å^3	3325.6(2)
Z	4
ρ_{calc}g/cm^3	1.463
μ/mm^{-1}	2.475
F(000)	1488.0
Crystal size/mm^3	0.18 × 0.12 × 0.1
Radiation	MoKα (λ = 0.71073)
2Θ range for data collection/deg	4.4 to 54.368
Index ranges	-23 ≤ h ≤ 23, -25 ≤ k ≤ 25, -11 ≤ l ≤ 11
Reflections collected	123401
Independent reflections	3695 [R_{int} = 0.0442, R_{sigma} = 0.0136]
Data/restraints/parameters	3695/68/292
Goodness-of-fit on F^2	1.047
Final R indexes [I ≥ 2σ (I)]	R_1 = 0.0440, wR_2 = 0.1210
Final R indexes [all data]	R_1 = 0.0561, wR_2 = 0.1294
Largest diff. peak/hole / e Å^{-3}	0.86/-0.91

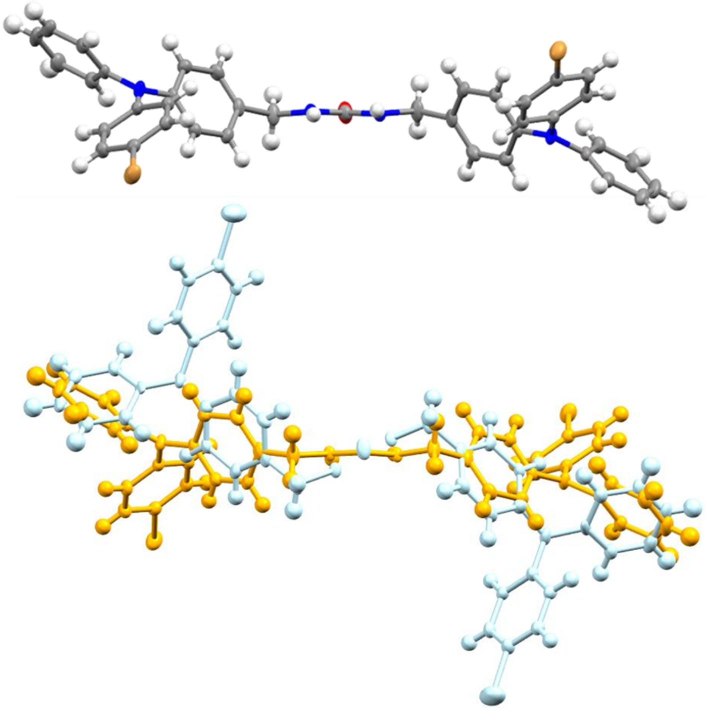

Figure S9. Thermal ellipsoid view of **3** at 50% probability (above) and disordered structure of molecule (below). For the disordered structure, two orientations are present on the C_2 axis with the CO on the two-fold axis in common with both. The orange structure is the major component at 91% vs. 9% for the other.

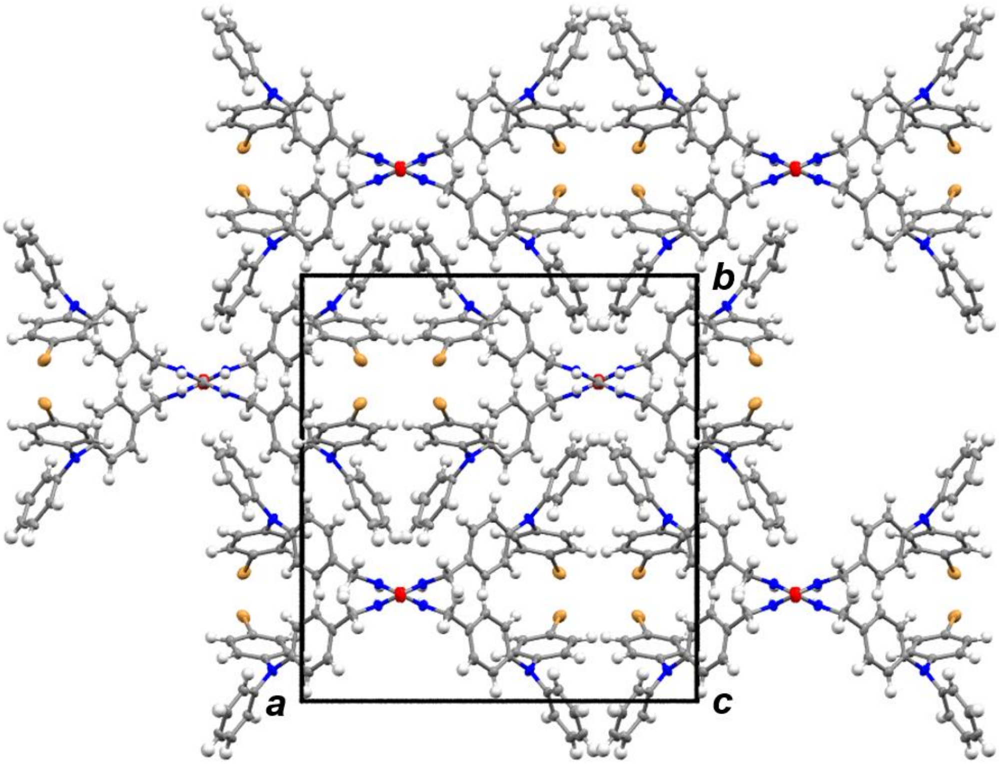

Figure S10. Crystal packing of **3** looking down the *c*-axis. Disorder was omitted for clarity.

Powder X-ray Diffraction (PXRD)

PXRD data was collected on a Rigaku D/Max-2100 powder X-ray diffractometer using Cu Kα radiation. The step can covered an angular range of 10-50˚ 2*Θ* in steps of 0.02˚.

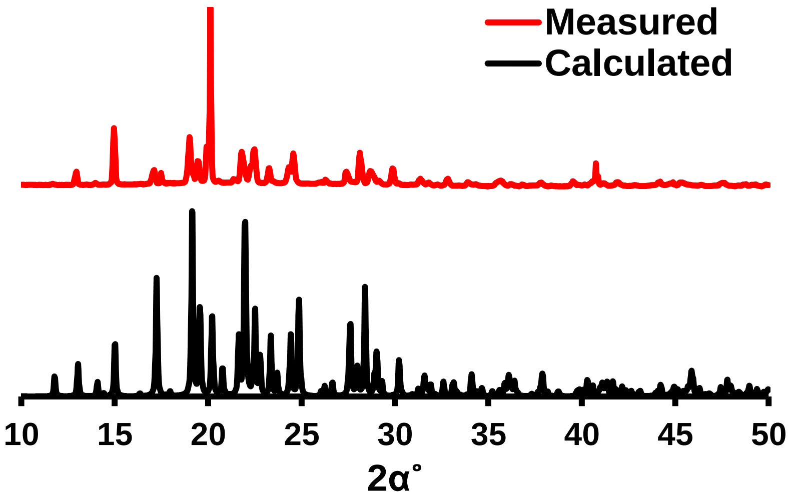

Figure S11. PXRD of **3**.

Absorbance Measurements

UV/Vis data was collected on either a Perkin Elmer Lambda 35 UV/vis spectrometer with UV Winlab software or a SoftMax M2 spectrometer (solid and solution, respectively). Spectra were recorded from 270-550 nm at 1 nm steps at room temperature. 10 μM concentrations were used for solution samples, unless otherwise noted. For solid samples, diffuse reflectance data was gathered initially and sequentially converted into absorbance data.

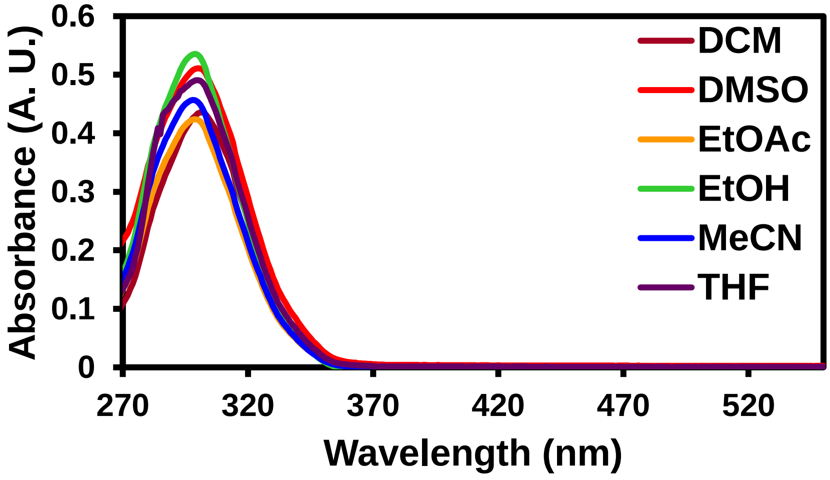

Figure S12. UV/Vis absorption spectrum of **1** in different solvents. Concentrations of 20 μM were used.

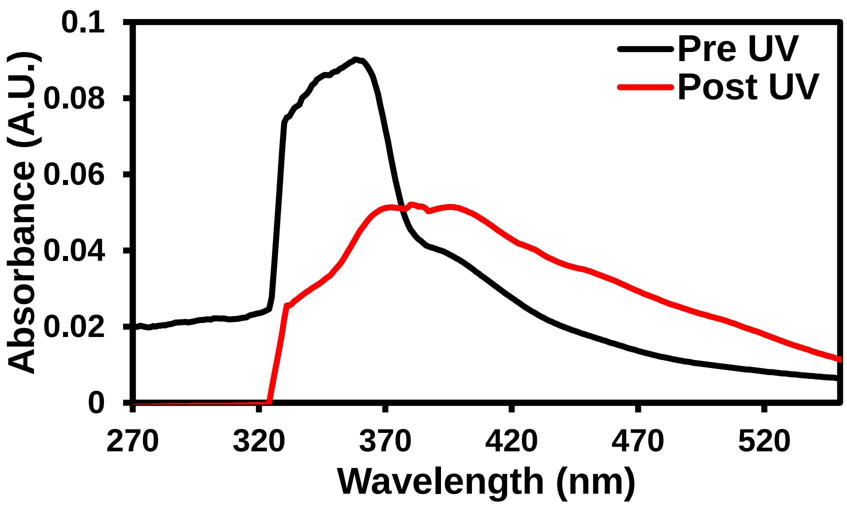

Figure S13. Absorbance for **3** pre and post UV for the solid-state. Sample was irradiated for 4 hours before post measurement.

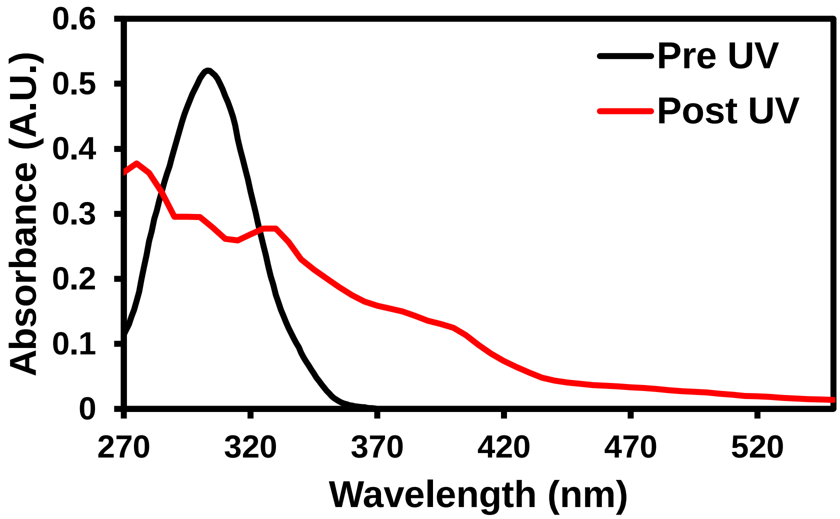

Figure S14. Absorbance for **3** pre and post UV in DCM (10 µM). Sample was irradiated for 4 hours before post UV measurement. Pre UV measurement was recorded in 1 nm steps. Post UV measurement was taken in 5 nm steps.

Emission Measurements

Emission data was collected on an Edinburgh FS5 instrument equipped with a 150 W continuous wave xenon lamp source for excitation. Excitations were performed at the λ_{max} of absorbance. Spectra were gathered from X-800 nm at 1 nm steps and are an average of three measurements (X = 25 nm red-shifted from excitation wavelength). Measurements were performed at room temperature.

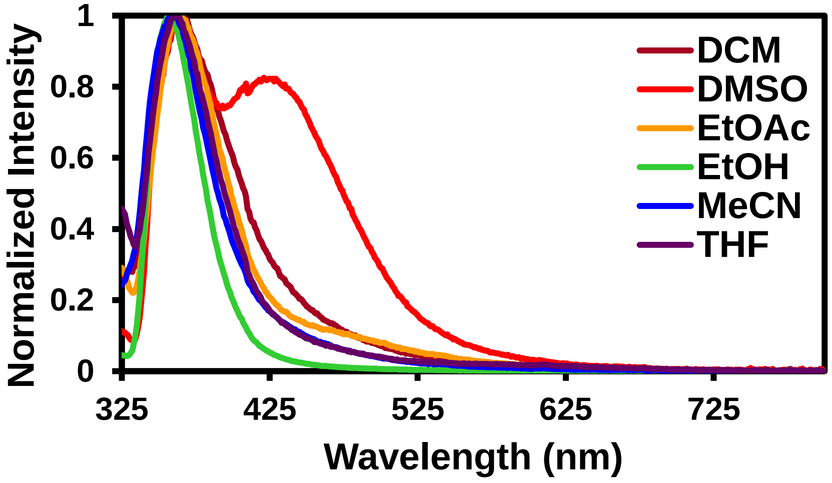

Figure S15. Emission spectrum of **1** (20 µM) in different solvents.

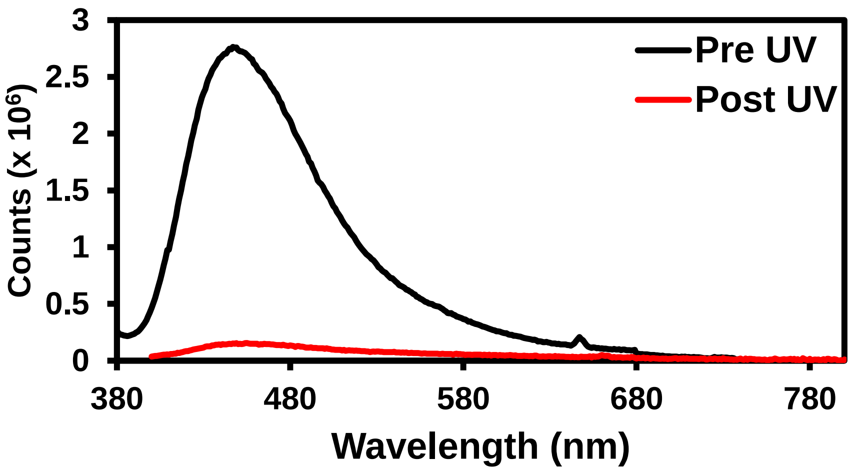

Figure S16. Emission for **3** pre and post UV for the solid-state. Sample was irradiated for 4 hours before post measurement.

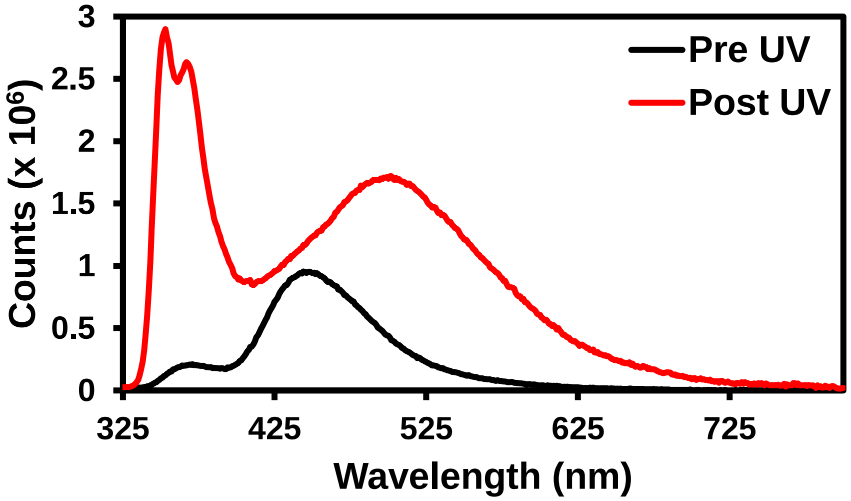

Figure S17. Emission for **3** pre and post UV in DCM (10 µM). Sample was irradiated for 4 hours before post UV measurement. Post UV measurement was irradiated at same λ_{max} as pre UV since no clear λ_{max} was shown in post UV absorbance.

Table S2. Photophysical data for **1**-**3**.

Compound	Solvent	$\lambda_{abs.}$[a]	ε (x 10^4)[b]	$\lambda_{ems.}$[c]	Stoke's Shift[d]
1	DCM	301	2.18	365	64
	THF	300	2.45	360	60
	EtOH	299	2.68	359	60
	EtOAc	299	2.12	365	66
	MeCN	298	2.28	358	60
	DMSO	300	2.56	365*, 427	65
2	DCM	304	5.11	365, 435*	131
	THF	302	5.47	366*, 444	64
	EtOH	301	4.95	361	60
	EtOAc	301	5.34	365	64
	MeCN	300	4.70	371, 451*	151
	DMSO	302	5.42	451	149
3	DCM	303	5.20	366, 449*	146
	THF	302	5.22	367*, 457	65
	EtOH	300	5.45	362*, 437	62
	EtOAc	301	5.53	364*, 453	63
	MeCN	300	5.01	497	197
	DMSO	302	5.41	369, 452*	150
	Solid State	358	--	447	89

[a] Peak position at largest absorption band in nm. [b] Molar absorptivity (M^{-1} x cm^{-1}). [c] Peak positions at largest emission bands in nm (largest denoted with * if applicable, excited at $\lambda_{abs.}$). [d] Stoke's shift in nm.

Diffused Ordered SpectroscopY (DOSY) NMR

Diffusion measurements were performed on a Bruker Avance IIIHD 400 MHz spectrometer using the vendor-supplied BPPE-LED[9] pulse sequence. 1 mM and 100 µM solutions of **3** in CD_3CN were prepared beforehand and spectra were recorded with a 25 ms diffusion delay and a 2.2 ms diffusion gradient.

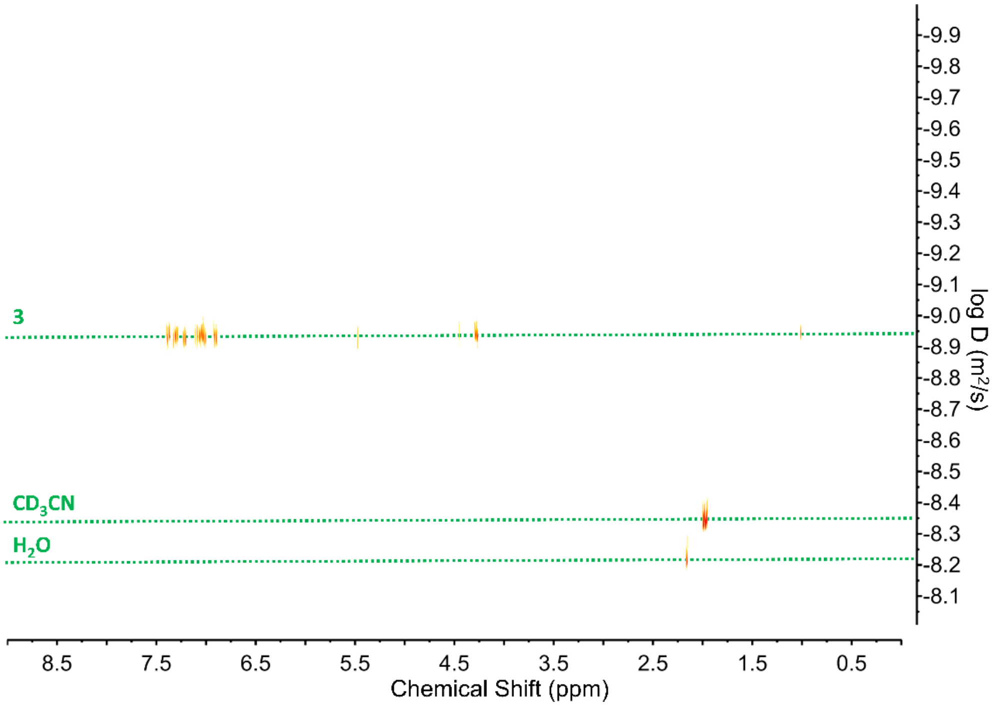

Figure S18. DOSY spectra of **3** as a 1 mM solution in CD_3CN.

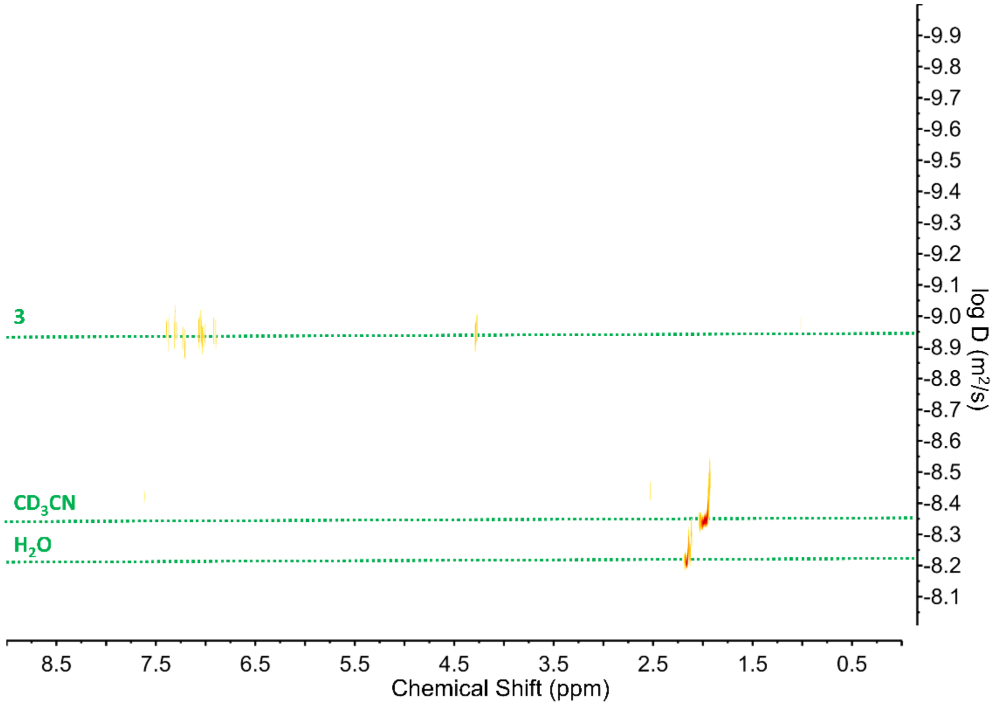

Figure S19. DOSY spectra of **3** as a 1 µM solution in CD_3CN.

$$r_{\text{solute}} = \frac{\boldsymbol{D}_{\text{MeCN}}\, \boldsymbol{r}_{\text{MeCN}}}{\boldsymbol{D}_{\text{solute}}}$$

Equation 1. Modified Stokes-Einstein equation to solve for the hydrodynamic radius of a solute. $\boldsymbol{D}_{\text{MeCN}}$ and $\boldsymbol{D}_{\text{solute}}$ are determined experimentally while $\boldsymbol{r}_{\text{MeCN}}$ is from a reference value.

Table S3. DOSY values for **3**.

Measurement	Compound	Diffusion Coefficient (x 10^{-9} m^2/s)	Hydrodynamic Radius (Å)
1 mM	CD_3CN	4.57	2.05[10]
	3	1.17	8.01[a]
100 µM	CD_3CN	4.47	2.05[10]
	3	1.12	8.18[a]
Crystal	3	N/A	5.83[b]

[a]Values calculated from Equation 1. [b]Radius of **3** calculated from crystal structure.

Lifetime Measurements

Lifetimes were measured using a Mini-τ lifetime spectrometer from Edinburgh Instruments equipped with either a 300 nm picosecond-pulsed-light-emitting diode (EPLED 300) or an EPLED 365 on 10 µM solutions. The lifetimes were recorded for the largest emission peak given in Table 1 and were recorded at room temperature. The decays for **2** and **3** were fit according to Equation S2 as either a bi- or triexponential function where τ and *B* are the lifetime and amplitude, respectively.

$$\mathrm{I(t)} \; = \int_{-\infty}^{t} \mathrm{IRF}(t') \sum_{\mathrm{i=1}}^{\mathrm{n}} B_{\mathrm{i}} e^{-\frac{t-t'}{\tau_i}} \, dt'$$

Equation S2. Fitting equation for fluorescence decay.

The amplitude-weighted average luminescent lifetimes τ_{av} were calculated using Equation S3. B_3 and τ_3 were only used for triexponential fits.

$$\langle \tau_{\mathrm{av}} \rangle = \frac{B_1\tau_1 + \; B_2\tau_2 + \; B_3\tau_3}{B_1 + \; B_2 + \; B_3}$$

Equation S3. Equation for amplitude-weighted average lifetime.

Table S4. Lifetimes for **2** and **3**.

Compound	Solvent	B_1	τ_1 (ns)	B_2	τ_2 (ns)	B_3	τ_3 (ns)	τ_{av} (ns)	X^2
2	DCM	0.1390	0.236	0.0310	1.727	0.0112	4.991	0.8	1.252
	DMSO	0.0253	3.084	0.0344	6.617			5.1	1.472
	EtOAc	0.6173	0.101	0.0037	1.180	0.0004	4.914	0.1	1.010
	EtOH	0.7673	0.087	0.0033	1.427	0.0004	6.443	0.1	1.098
	MeCN	0.0489	1.957	0.0289	4.855			3.0	1.302
	THF	0.8936	0.076	0.0041	1.283	0.0007	5.750	<0.1	1.112
3	DCM	0.0447	0.828	0.0355	3.281	0.0061	6.480	2.2	1.164
	DMSO	0.0512	2.090	0.0379	6.719			4.1	1.445
	EtOAc	0.7800	0.082	0.0046	1.698	0.0017	3.914	0.1	1.045
	EtOH	0.6104	0.104	0.0048	1.236	0.0007	4.500	0.1	1.117
	MeCN	0.0648	2.748	0.0033	4.480			2.8	1.307
	THF	1.3075	0.059	0.0020	1.534	0.0005	5.491	<0.1	1.064
	Solid State	0.0728	0.392	0.0423	1.427	0.0086	3.282	1.0	1.168

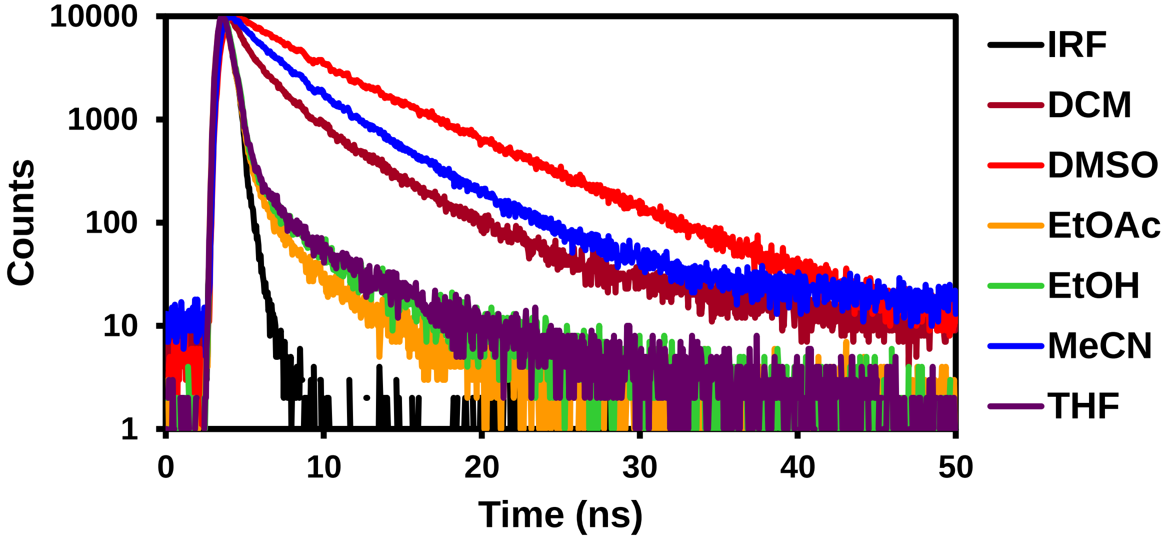

Figure S20. Lifetime data for **2** in different solvents.

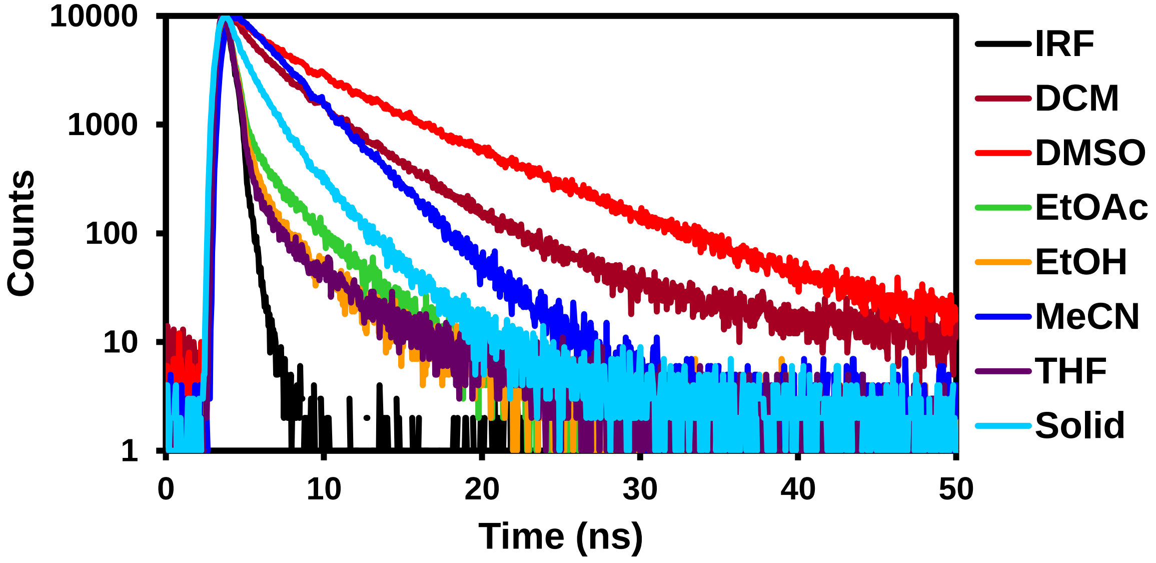

Figure S21. Lifetime data for **3** in different solvents and the solid-state.

EPR Measurements:

EPR measurements were carried out on a Bruker EMX plus equipped with a Bruker X-band microwave bridgehead and Xenon software (v 1.1b.66). All spectra were recorded at room temperature unless specified. All spectra were recorded at a power of 1.589 mW with a modulation amplitude of 2.0 G. The double integration to obtain peak areas was performed in the Xenon software. Samples were sealed under argon and UV-irradiated in Norell Suprasil Quartz EPR tubes.

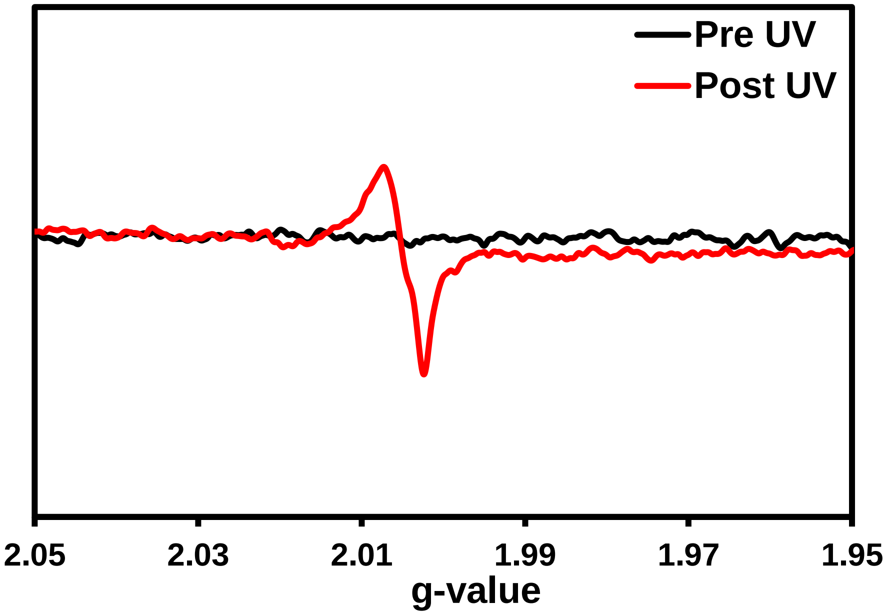

Figure S22. EPR spectra of **3** as a 1.09 mM solution in DCM. Sample was irradiated for 6 hours before measurement.

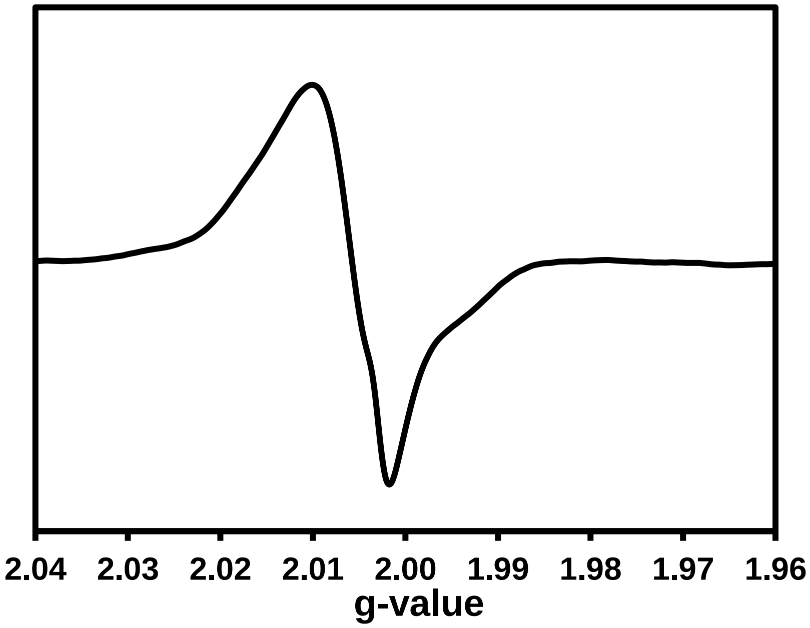

Figure S23. EPR spectra of triply recrystallized **3**. Sample was irradiated for 6 hours before measurement.

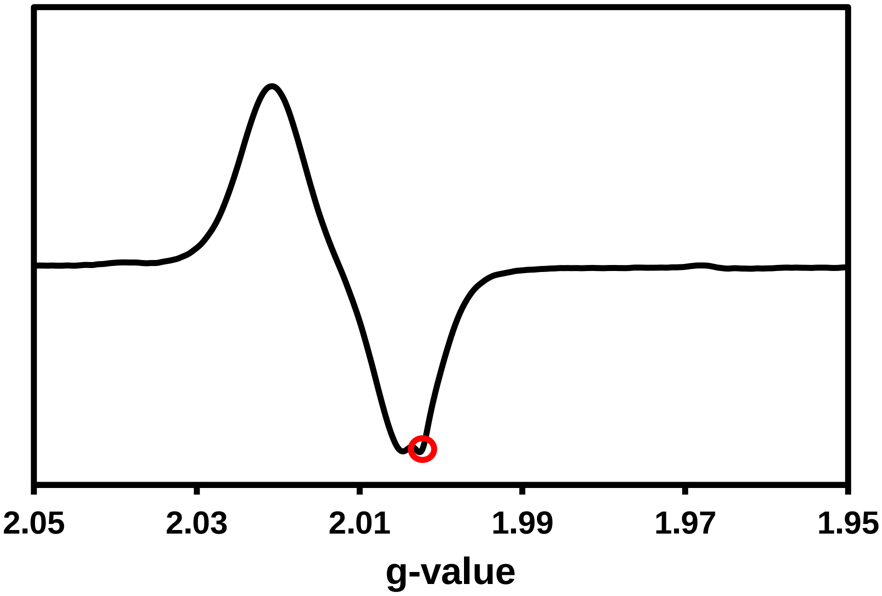

Figure S24. EPR spectra of 1 mM solution of *tris*(4-bromophenyl)ammoniumyl hexachloroantimonate (Magic Blue) in DCM. Quartz impurity from EPR tube at g = 2.002 is marked by red circle.

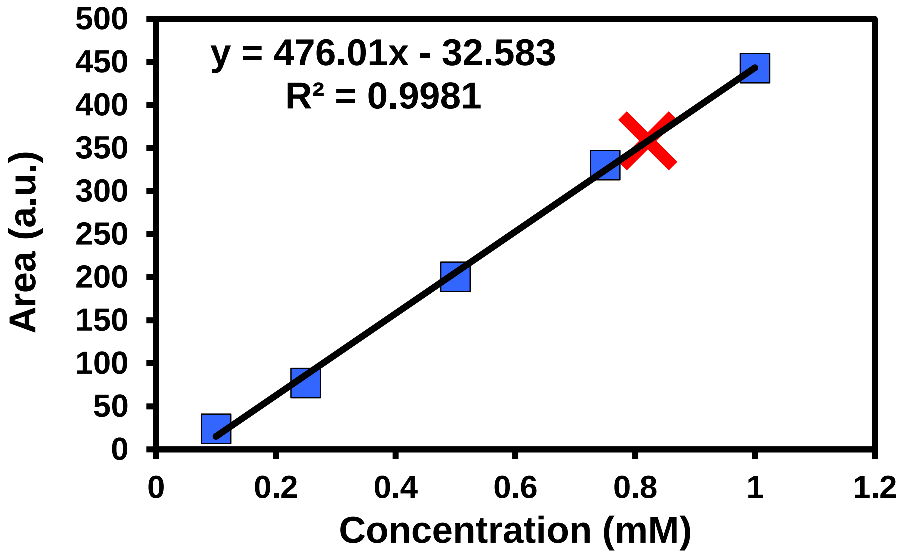

Figure S25. Radical concentration determination. The Magic Blue calibration curve is plotted and overlaid with the area and determined concentration of **3** in the solid-state (labeled as a red X). The solid-state measurement was taken as the average of the last four data point from Figure 4D from the max radical concentration determination experiment.

NMR Spectra Pre and Post UV

NMR spectra were taken on a Bruker Avance III-HD 300 MHz spectrometer. Samples were UV-irradiated 6 hours before measurement unless otherwise indicated.

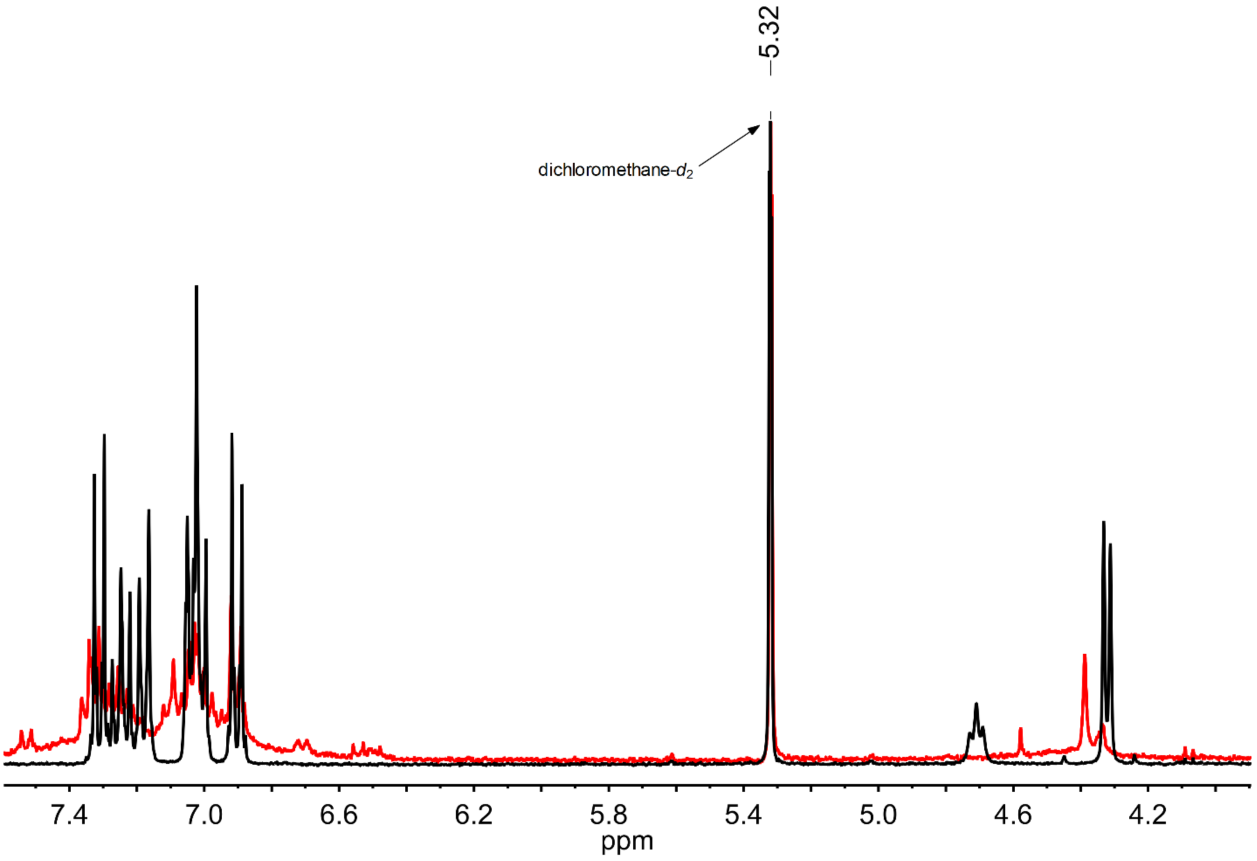

Figure S26. ^{1}H NMR of **3** (CD_2Cl_2, 300 MHz) as a solution in DCM pre (black) and post (red) UV irradiation. Significant changes were observed after irradiation.

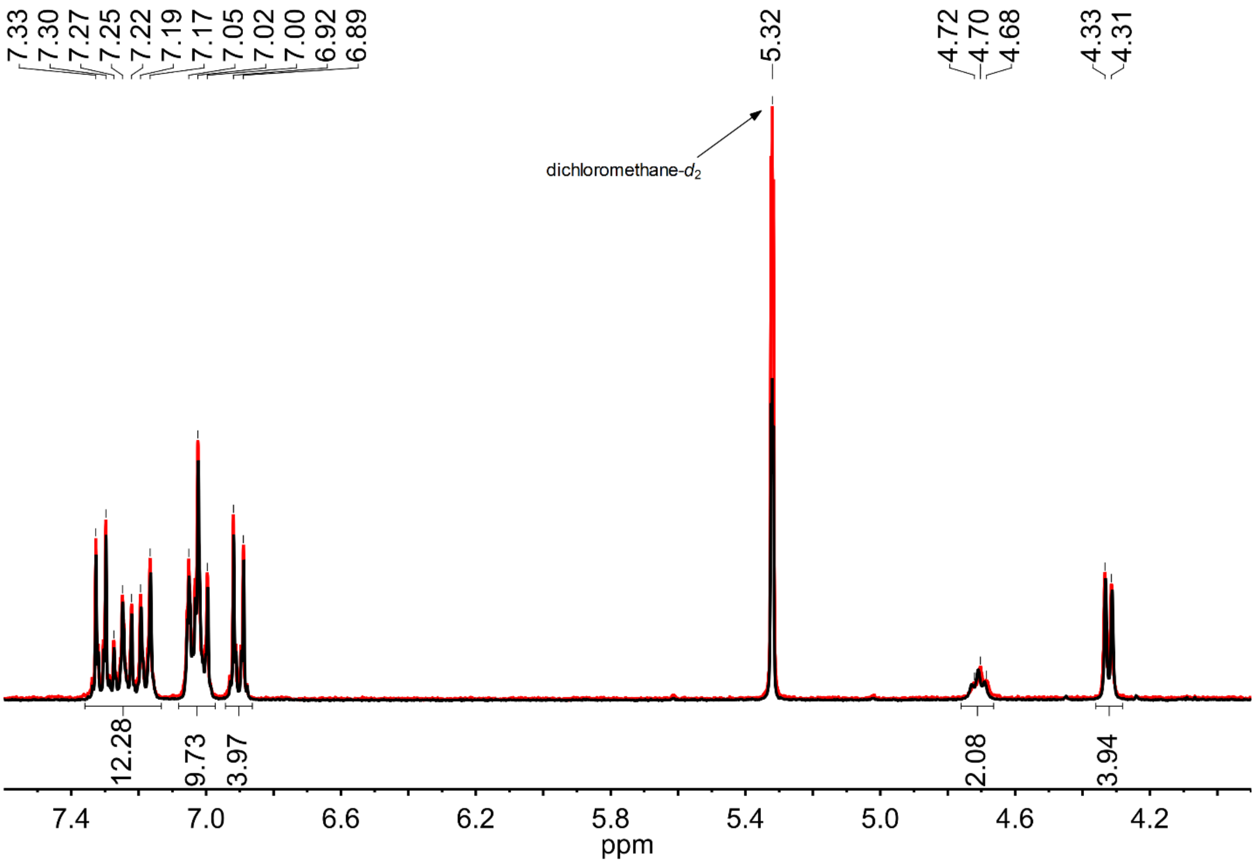

Figure S27. ^{1}H NMR of **3** (CD_2Cl_2, 300 MHz) for solid sample after decay of radical signal. Original radical was made from 1 hour of UV-irradiation. Sample was redissolved before measurement. No changes were observed upon dissolution. Peaks and integrals are for the redissolved sample (red). Black is for comparison represents an unirradiated sample.

Infrared Measurements (FT-IR)

All IR analysis were performed using a Perkin Elmer 100 IR Spectrometer. Spectra were taken from 650-4000 cm^{-1}.

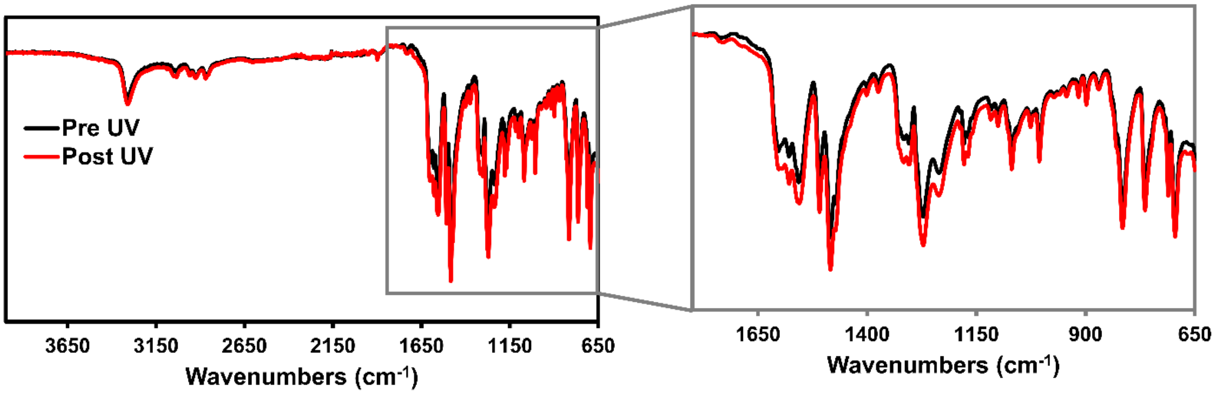

Figure S28. IR spectra for **3** pre and post UV for the solid-state. Sample was irradiated for 4 hours before post measurement.

Cyclic Voltammetry (CV)

Measurements were carried out in dichloromethane using a WaveDriver 20 Bipotentiostat combined with Aftermath software. Solutions contained 100 mM $(n\text{-Bu})_4N^+PF_6^-$ and 1 mM solute. Measurements were done in an H cell equipped with a SCE reference, platinum wire counter, and glassy carbon working electrodes. Measurements were conducted at a potential rate of 100 mV/s unless otherwise noted.

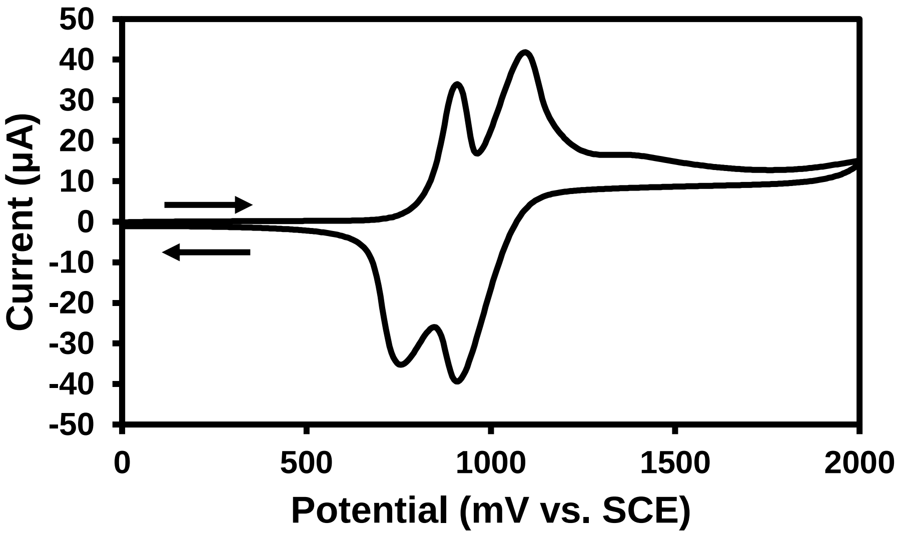

Figure S29. Oxidative cyclic voltammetry for **2**. Scanning rate was 40 mV/s instead of 100 mV/s.

Bulk Electrolysis

Measurements were carried out in dichloromethane using a WaveDriver 20 Bipotentiostat combined with Aftermath software. Cell was set up inside a nitrogen filled glovebox in a divided cell that contained an anode in a secondary container separated from the cathodic area by a glass frit. High surface RVC electrodes were used as both the working and counter electrodes while an SCE was used as the reference. The solution of **3** contained 10 mL of a 100 mM (n-Bu)$_4$N$^+$PF$_6^-$ and 1 mM solute unless otherwise noted. The solution was continuously stirred at 400 rpm during the experiment. [11]

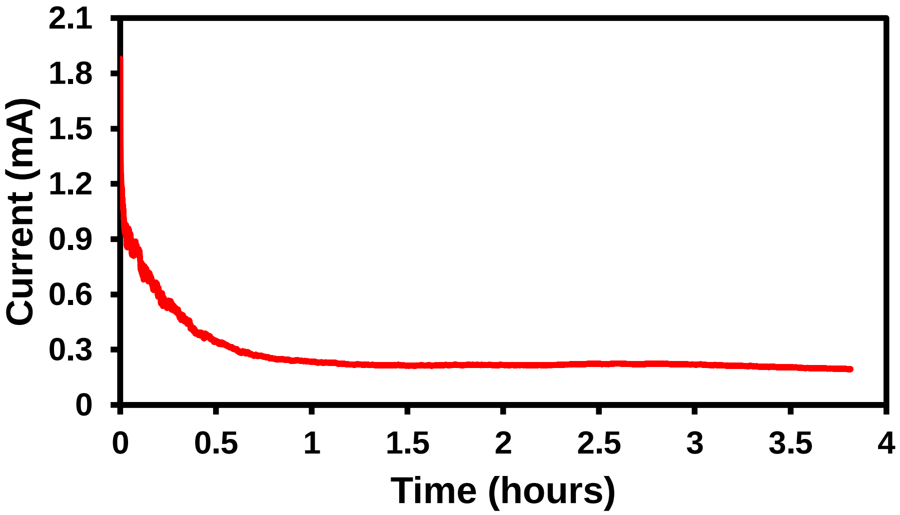

Figure S30. Electrolysis of **3** over time. 5 mL of a 0.5 mM solution of **3** was used instead of the standard conditions. Current was allowed to run down to 1% of its initial value. Potential was held at 1.25 V versus SCE. Total area under the curve was found to be 977.2 mC which amounts to 4 total electrons for **3** according to equation S4.

$$n = \frac{Q}{Fz}$$

Equation S4. Faraday's law of electrolysis where n is the amount of **3** in mols, Q is the amount of charge in columbs, F is Faraday's constant in C/mol, and z is the number of electrons.

Emission Quenching

Emission data was collected on an Edinburgh FS5 instrument equipped with a 150 W continuous wave xenon lamp source for excitation. Excitations were performed at the λ_{max} of absorbance (303 nm). Spectra were gathered from 325-800 nm at 1 nm steps and are an average of three measurements. Measurements were performed at room temperature.

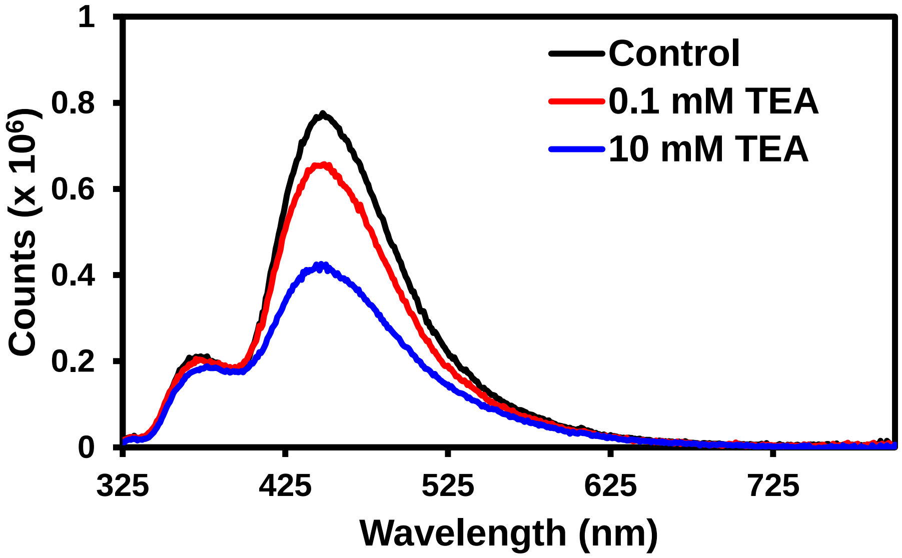

Figure S31. Emission for **3** (10 µM in dichloromethane) with different concentrations of triethylamine (TEA).

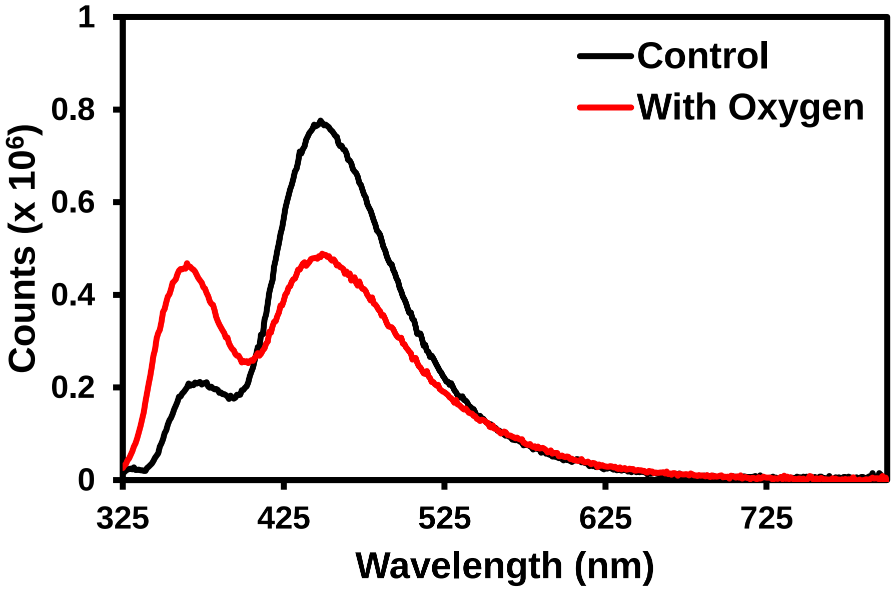

Figure S31. Emission for **3** (10 μM) in degassed dichloromethane (control) and oxygenated dichloromethane (with oxygen).

References

1. Z. Li, Q. Dong, B. Xu, H. Li, S. Wen, J. Pei, S. Yao, H. Lu, P. Li, and W. Tian, *Sol. Energy Mater. Sol. Cells*, 2011, **95**, 2272.

2. H. Tian, X. Yang, R. Chen, R. Zhang, A. Hagfeldt, and L. Sun, *J. Phys. Chem. C*, 2008, **112**, 11023.

3. G. G. Dubinina, R. S. Price, K. A. Abboud, G. Wicks, P. Wnuk, Y. Stephaneko, M. Drobizhev, A. Rebane, and K. S. Schanze, *J. Am. Chem. Soc.*, 2012, **134**, 19346.

4. *APEX3 version 2016.5−0, SAINT + Version 8.37A*; Bruker AXS, Inc.: Madison, WI, 2016.

5. SADABS-2016/2 L. Krause, R. Herbst-Irmer, G. M. Sheldrick, and D. Stalke, *J. Appl. Cryst.*, 2015, **48**, 3.

6. SHELXT G. M. Sheldrick, *Acta Crystallogr. A*, 2015, **A71**, 3; SHELXL G. M. Sheldrick, *Acta Crystallogr. C*, 2015, **C71**, 3.

7. O. V. Dolomanov, L. J. Bourhis, R. J. Gildea, J. A. K. Howard, and H. Puschmann, *J. Appl. Crystallogr*., 2009, **42**, 339.

8. A. L. Spek, *Acta Cryst. D*, 2009, **D65**, 148; Platon Homepage www.cryst.chem.uu.nl/platon/ (accessed Jul 26, 2018).

9. D. Wu, A. Chen, and C. S. Johnson, *J. Magn. Reson.*, 1995, **115**, 260.

10. J. Zhang and J. Jonas, *J. Phys. Chem.*, 1993, **97**, 8812.

11. D. M. M. M. Dissanayake and A. K. Vannucci, *ACS Substainable Chem. Eng.*, 2018, **6**, 690.
